# Supplementary material for: From stable Sb- and Bi-centered radicals to a compound with a Ga=Sb double bond
Source: Nat Commun. 2018 Jan 8;9:87. doi: 10.1038/s41467-017-02581-2 (PMC5758792; doi:10.1038/s41467-017-02581-2)
Supplement: Supplementary file 1 — Supplementary Information [file 41467_2017_2581_MOESM1_ESM.pdf]

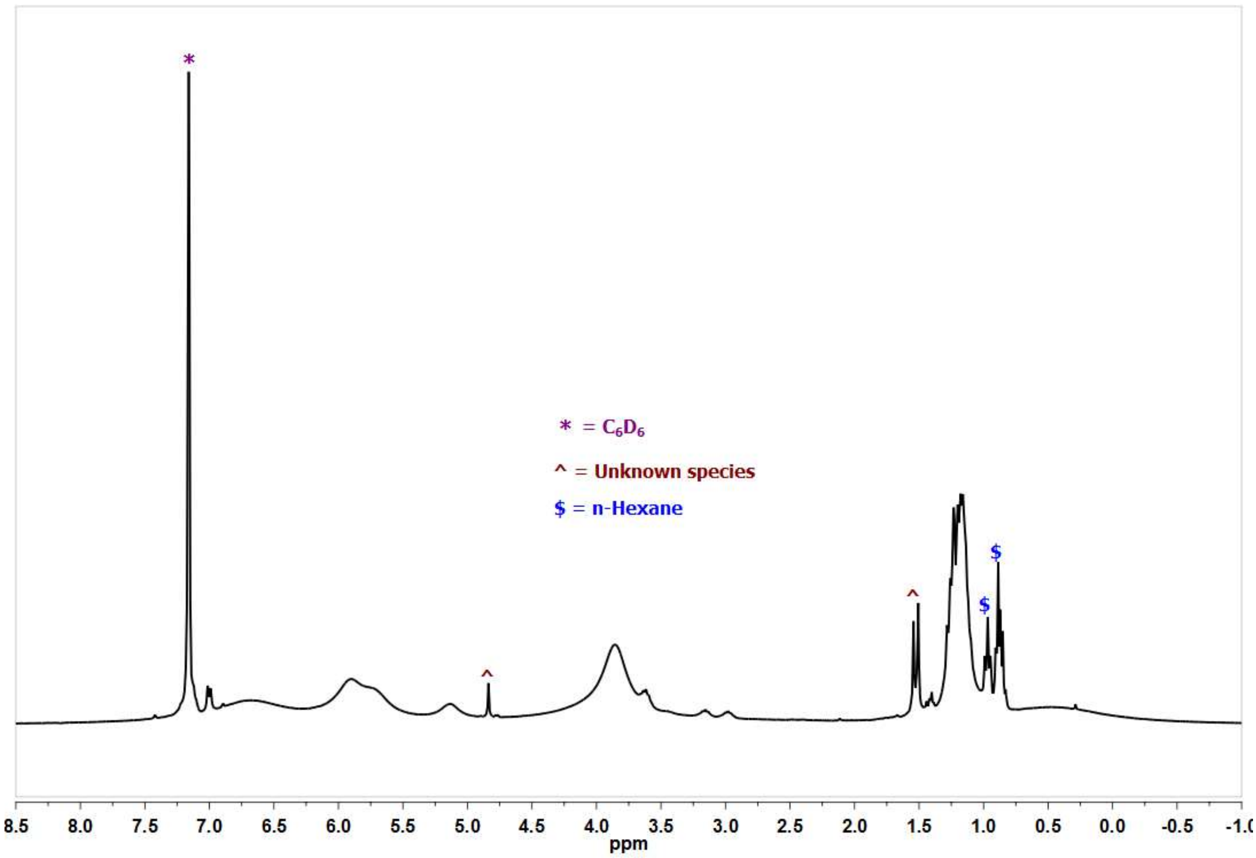

Supplementary Figure 1. ^{1}H NMR spectrum of $[L(Cl)Ga]_2Sb\cdot$ **1** in benzene-d_6 at room temperature.

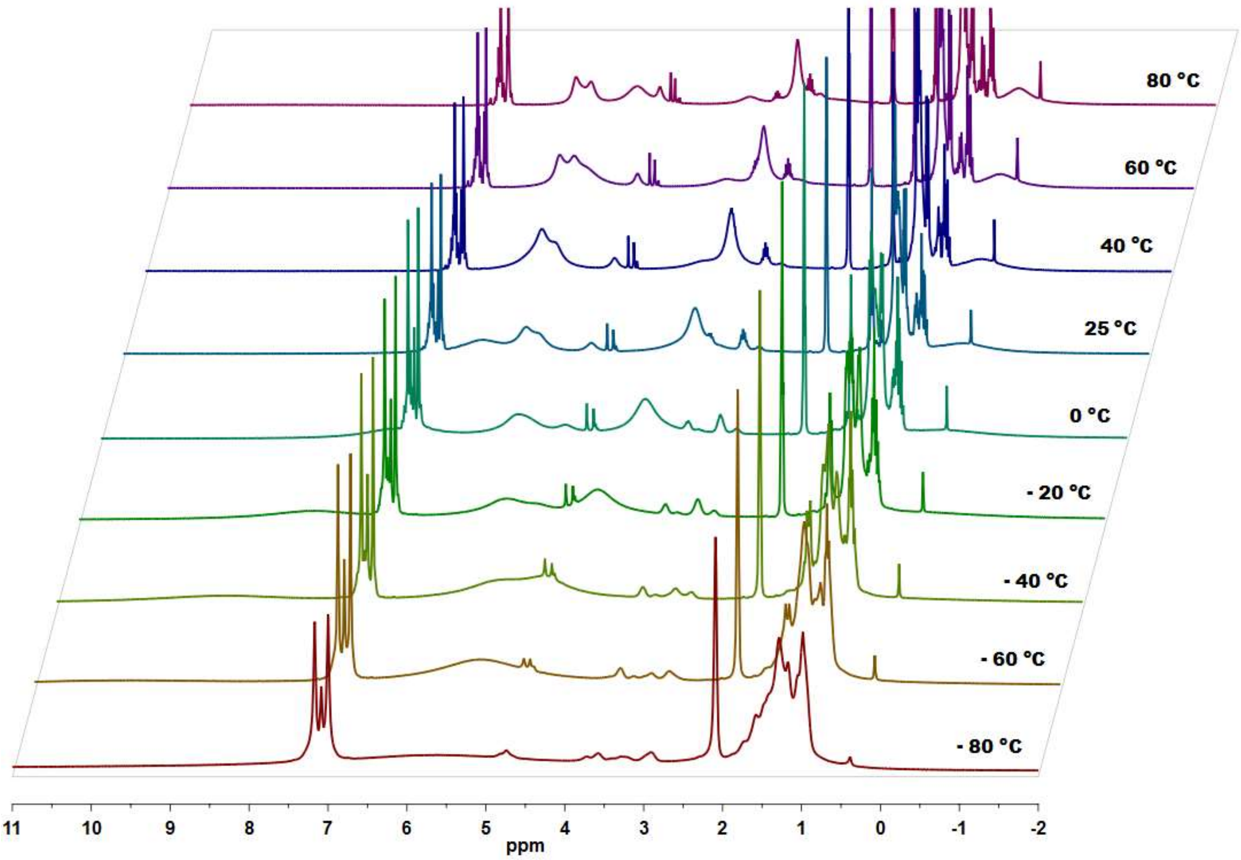

Supplementary Figure 2. Temperature-dependent ^{1}H NMR study of $[L(Cl)Ga]_2Sb\cdot$ **1** in toluene-d_8.

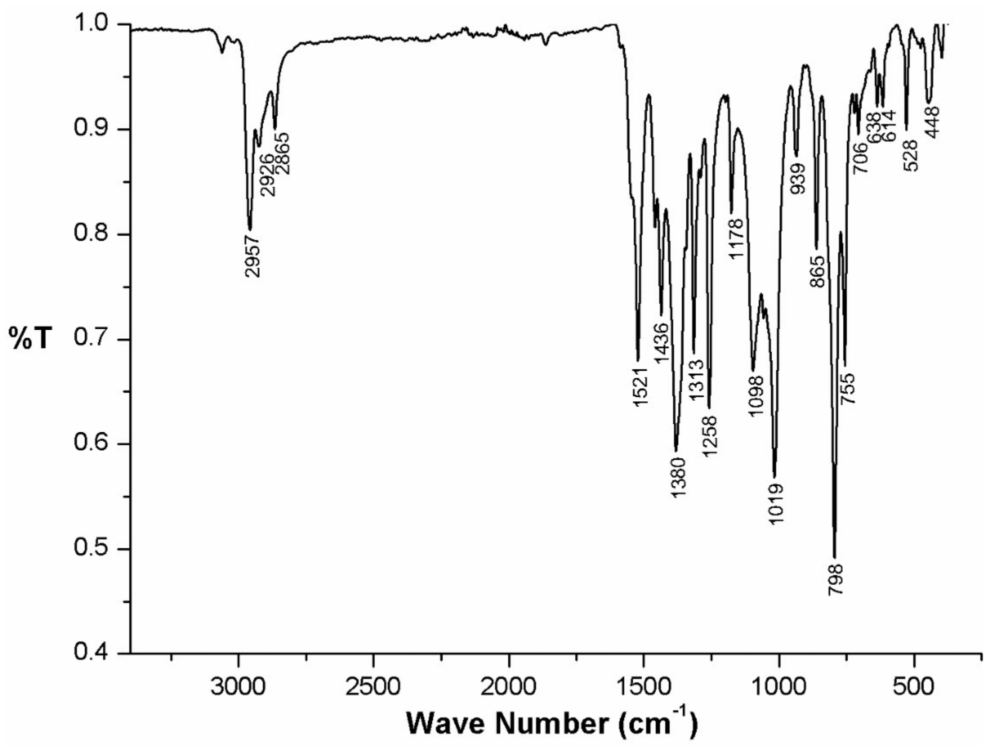

Supplementary Figure 3. ATR-IR spectrum of $[L(Cl)Ga]_2Sb$· **1**.

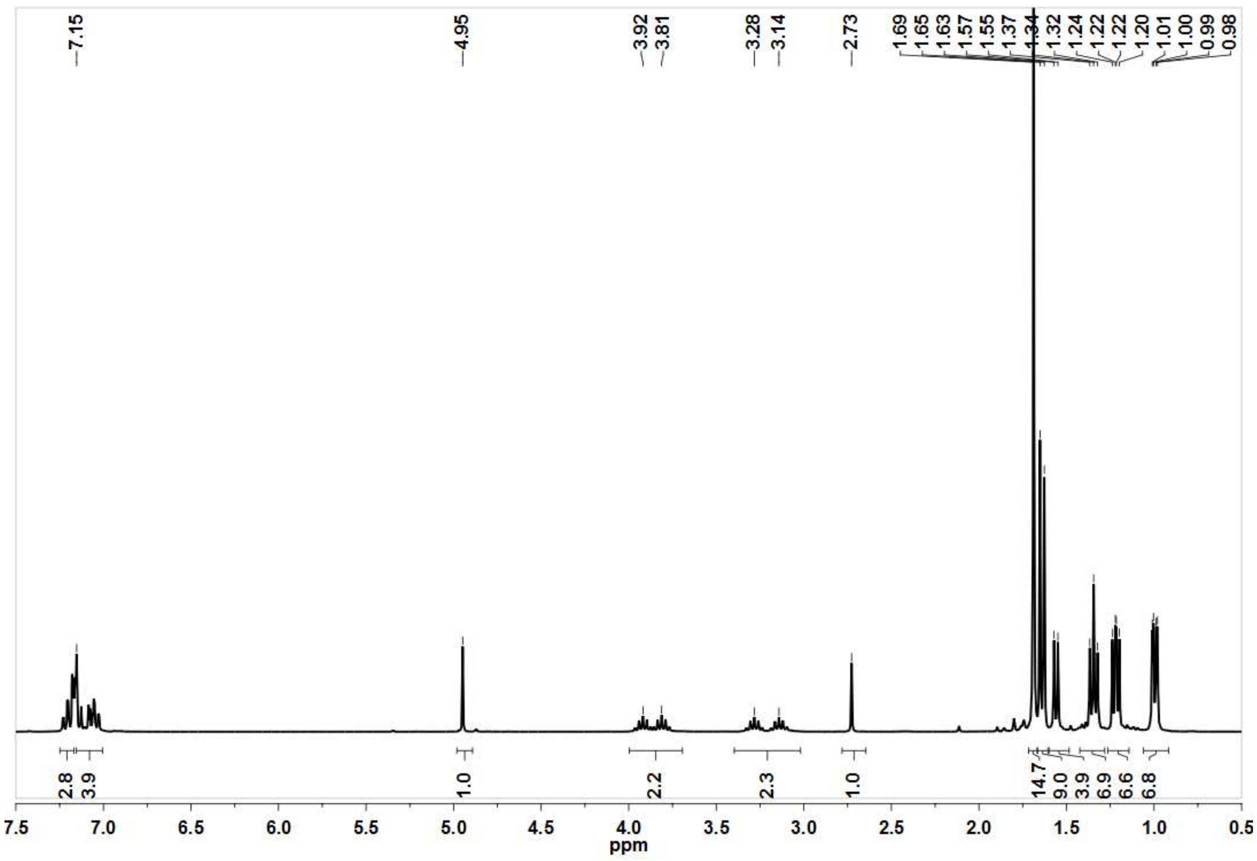

Supplementary Figure 4. ^{1}H NMR spectrum of L(Cl)GaSb(H)Cp* **2** in benzene-d_6 at room temperature.

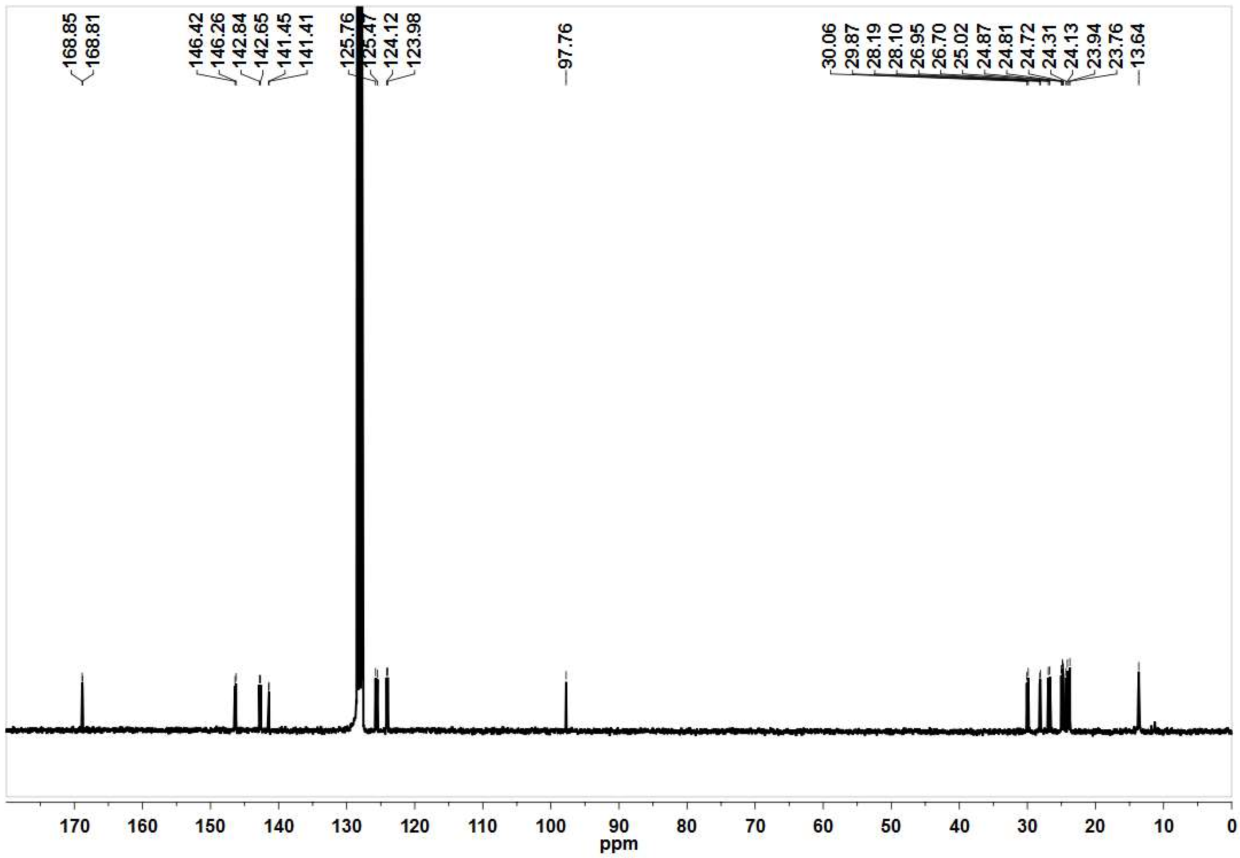

Supplementary Figure 5. ^{13}C NMR spectrum of L(Cl)GaSb(H)Cp* **2** in benzene-d_6 at room temperature.

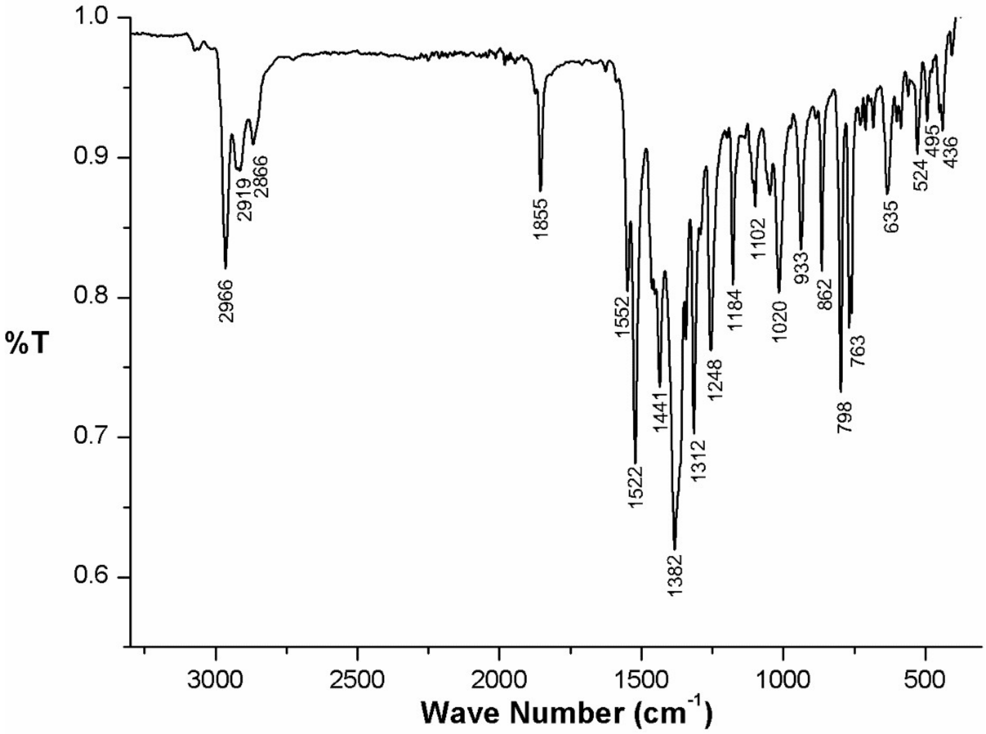

Supplementary Figure 6. ATR-IR spectrum of L(Cl)GaSb(H)Cp* **2**.

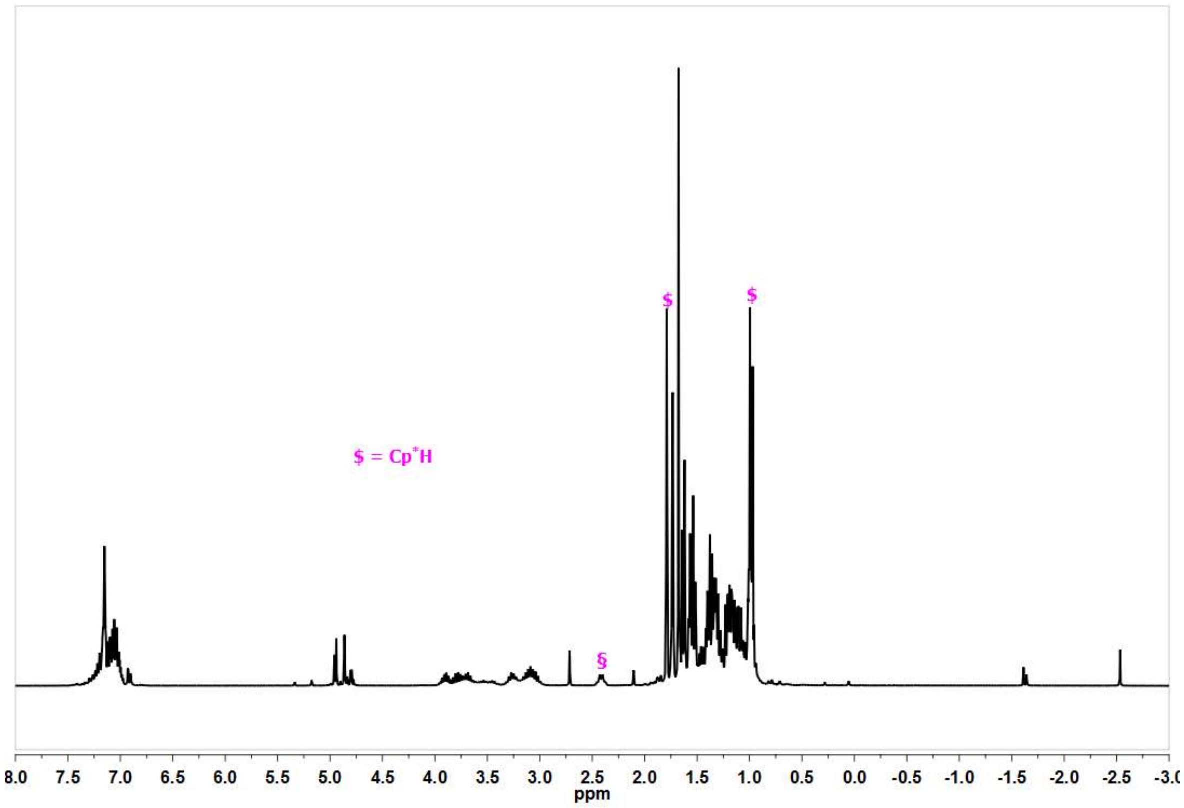

Supplementary Figure 7. ^{1}H NMR spectrum of L(Cl)GaSb(H)Cp* **2** in benzene-d_6 after stirring at room temperature for 3 days.

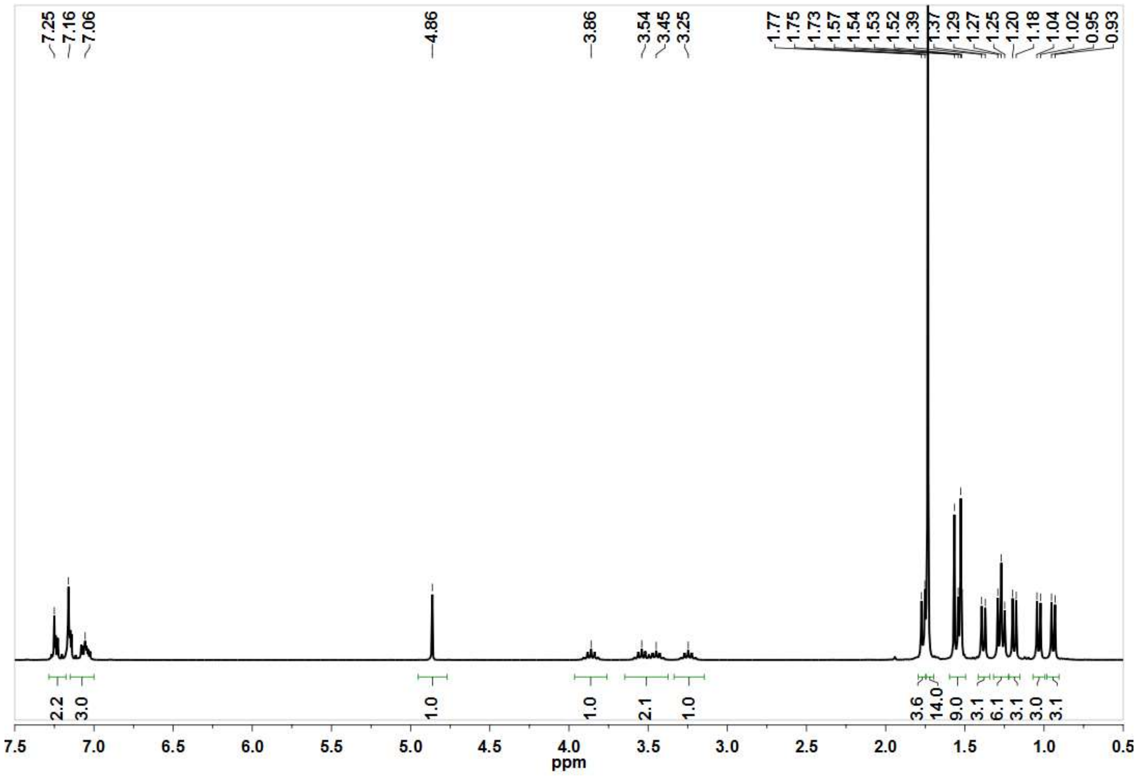

Supplementary Figure 8. ^{1}H NMR spectrum of L(Cl)GaSb(Cl)Cp* **3** in benzene-d_6 at room temperature.

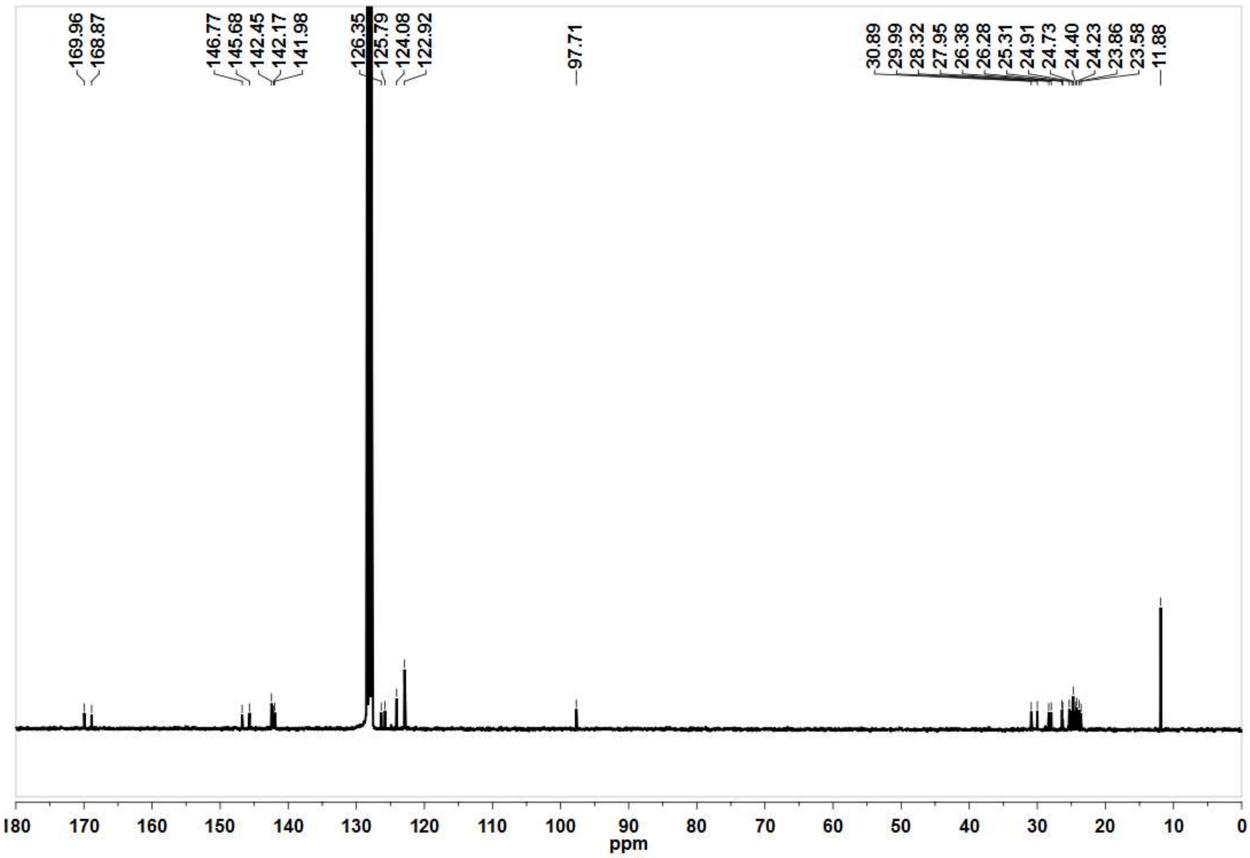

Supplementary Figure 9. ^{13}C NMR spectrum of L(Cl)GaSb(Cl)Cp* **3** in benzene-d_6 at room temperature.

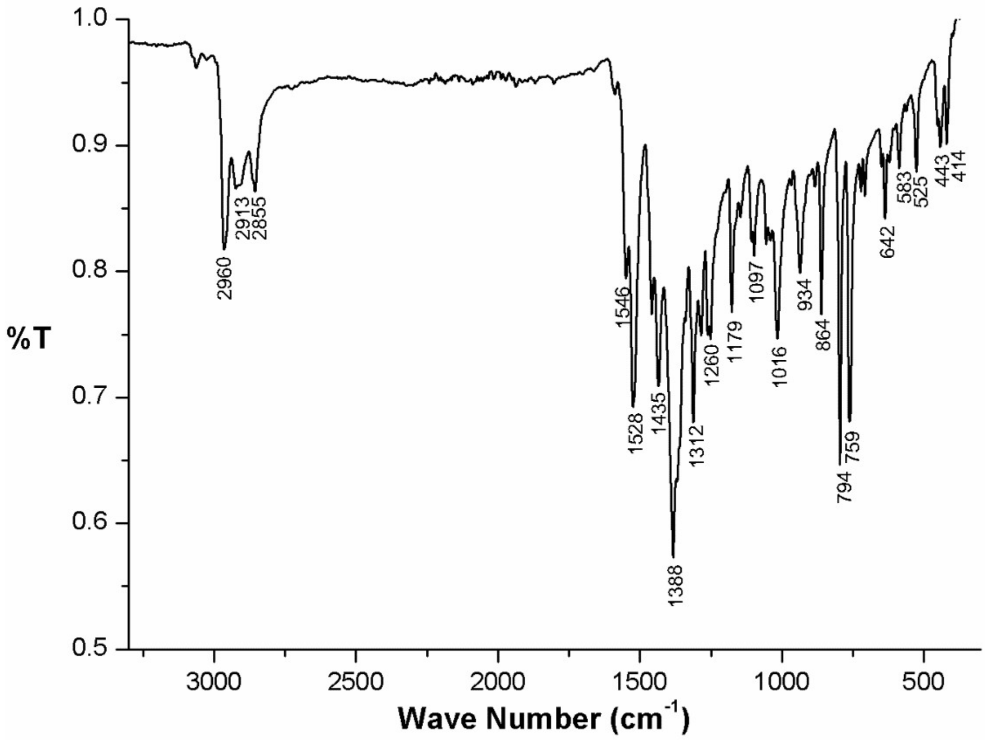

Supplementary Figure 10. ATR-IR spectrum of L(Cl)GaSb(Cl)Cp* **3**.

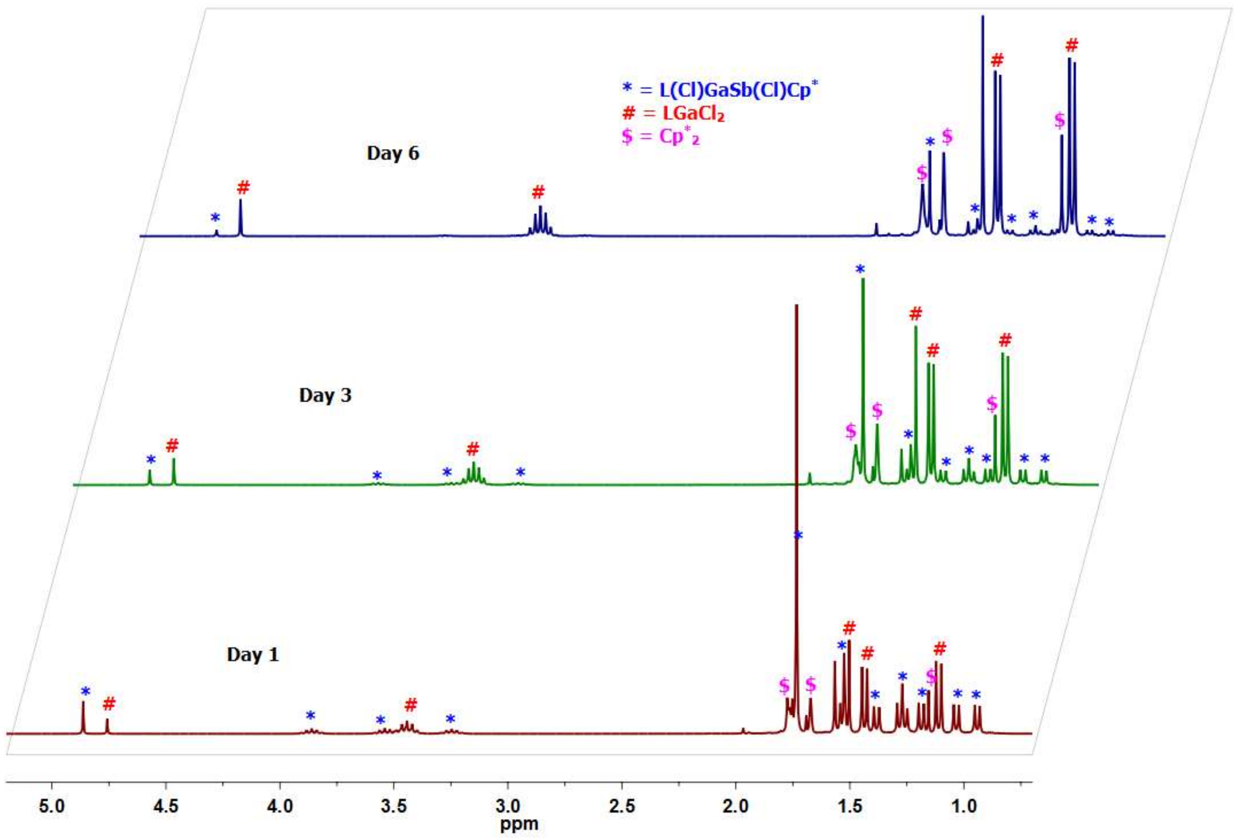

Supplementary Figure 11. ^{1}H NMR spectra of L(Cl)GaSb(Cl)Cp* **3** in benzene-d_6 after stirring at r.t. for 1, 3 and 6 days.

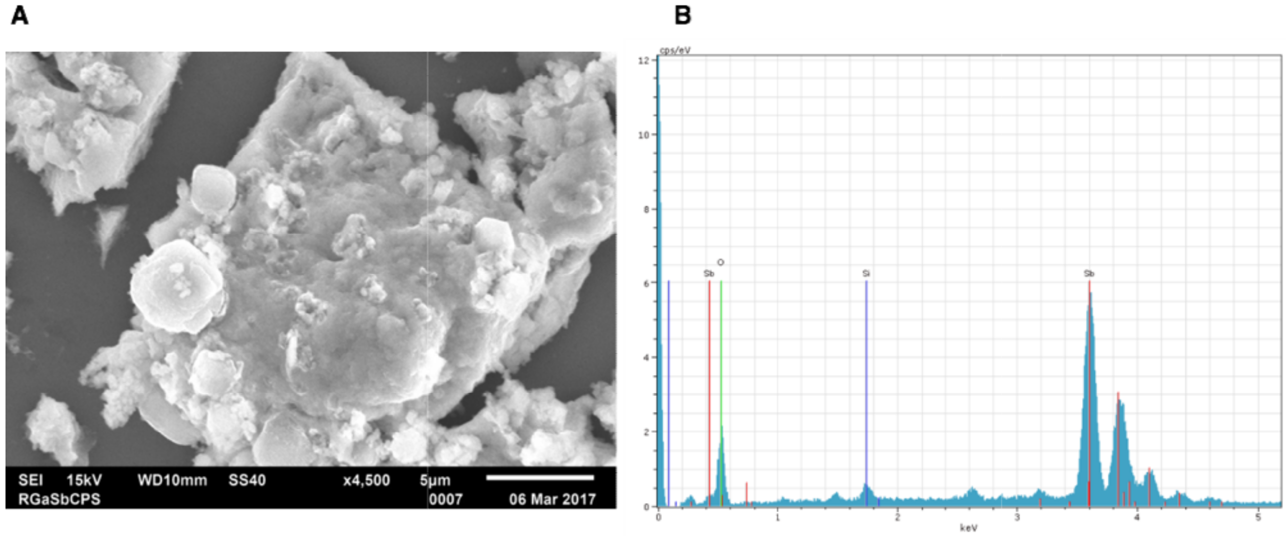

Supplementary Figure 12. SEM picture (A) and EDX spectrum (B) of the Sb metal formed from compound L(Cl)GaSb(Cl)Cp* **3** at room temperature.

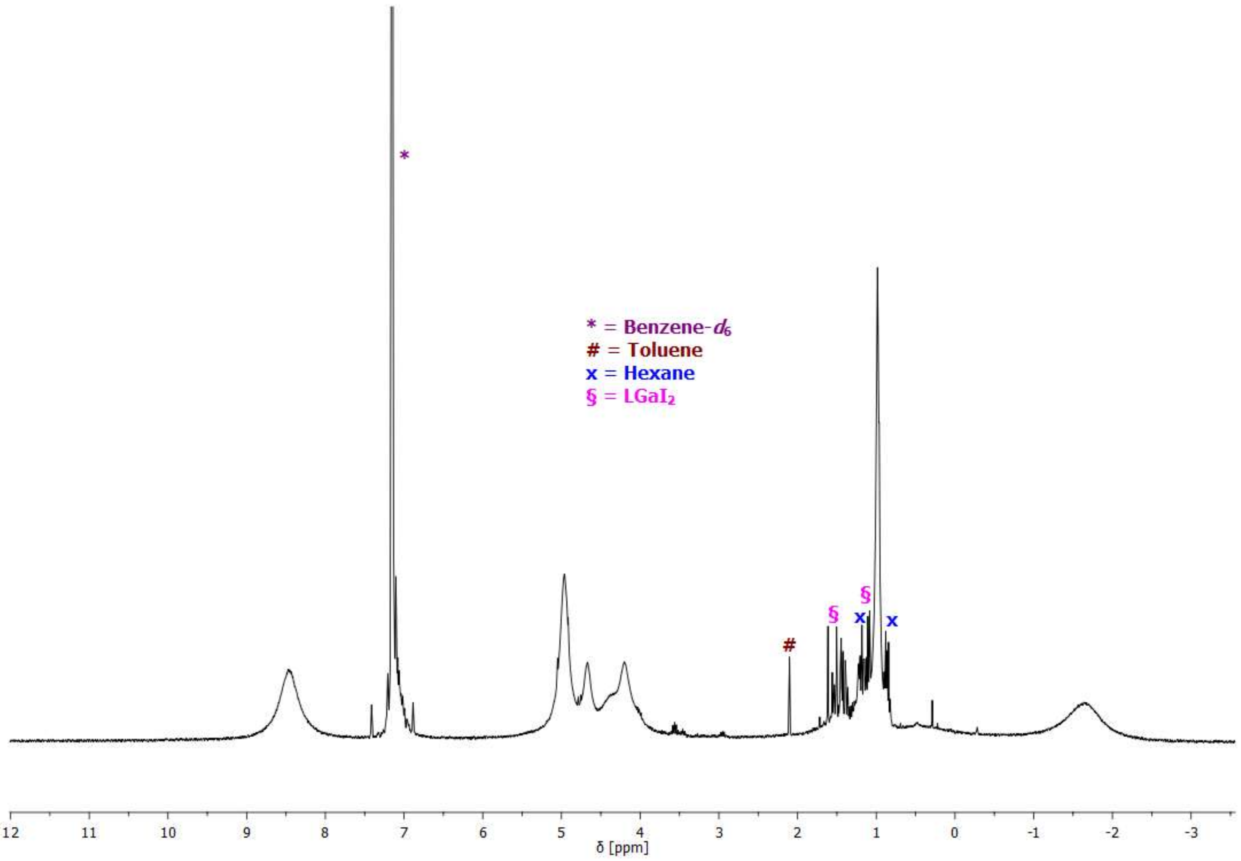

Supplementary Figure 13. ^{1}H NMR spectrum of $[L(I)Ga]_2Bi\cdot$ **4** in toluene-d_8 at room temperature.

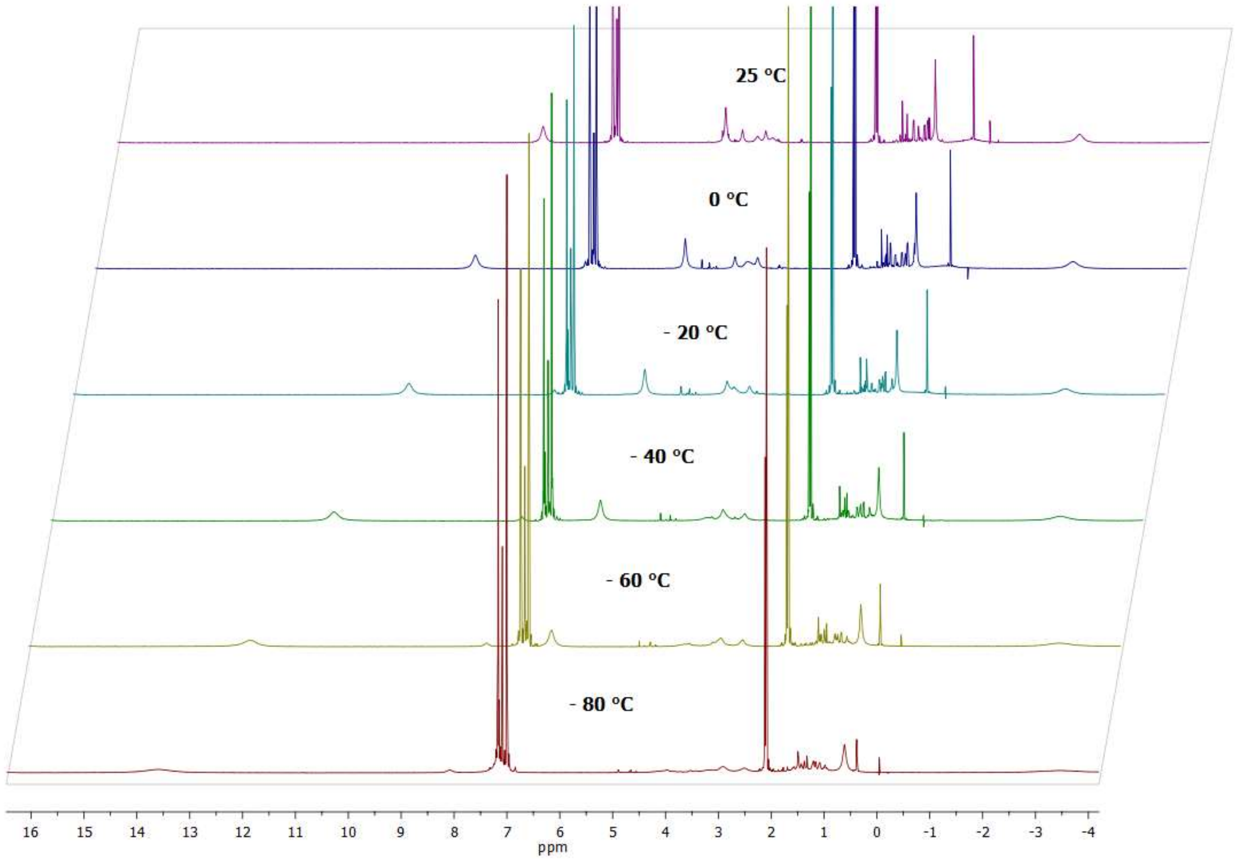

Supplementary Figure 14. Temperature-dependent ^{1}H NMR study of $[L(I)Ga]_2Bi\cdot$ **4** in toluene-d_8.

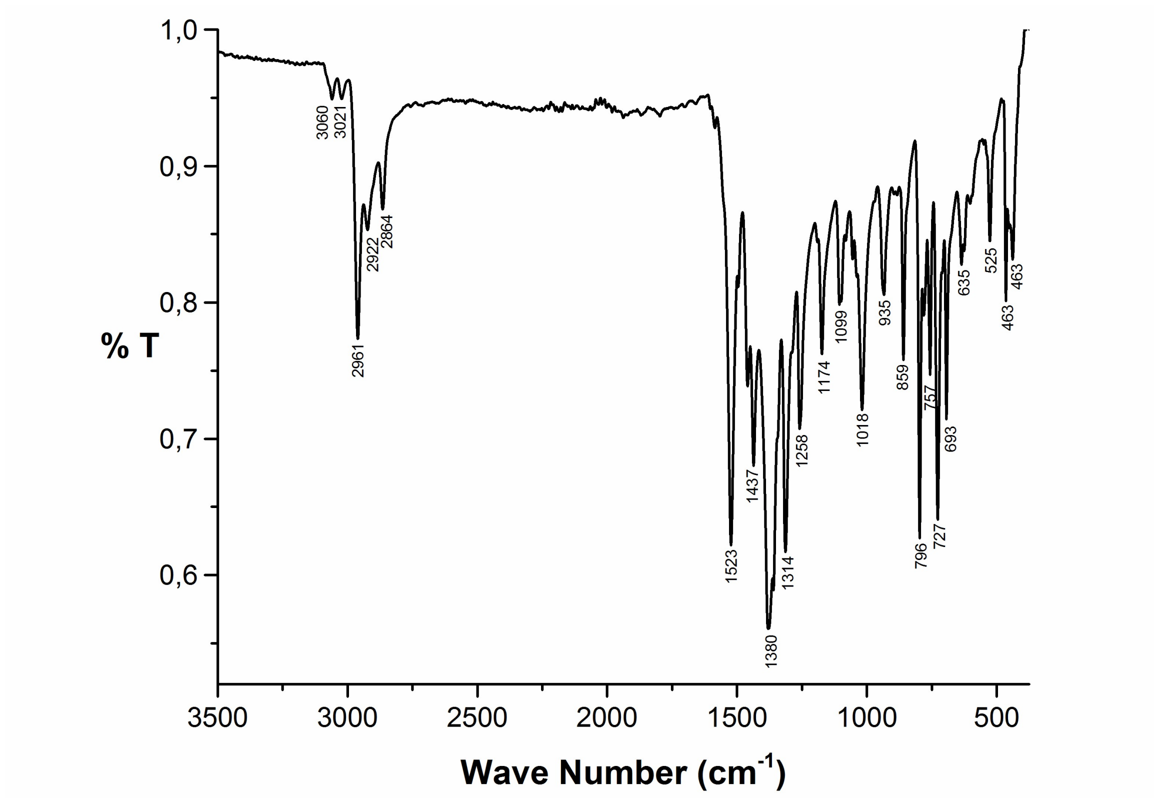

Supplementary Figure 15. ATR-IR spectrum of $[L(I)Ga]_2Bi\cdot$ **4**.

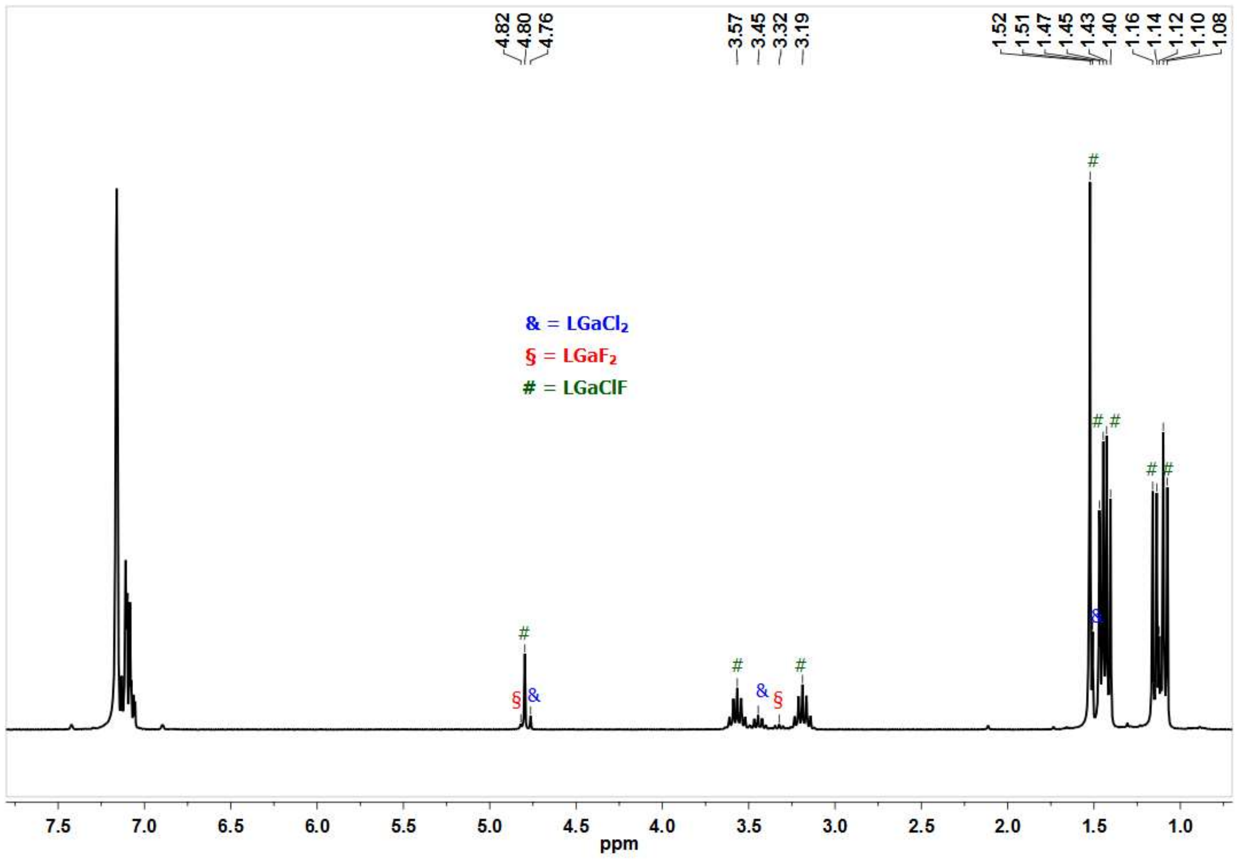

Supplementary Figure 16. The ^{1}H NMR spectrum of LGaClF in benzene-d_6 from the reaction of $[L(Cl)Ga]_2Sb\cdot$ **1** with $NOBF_4$.

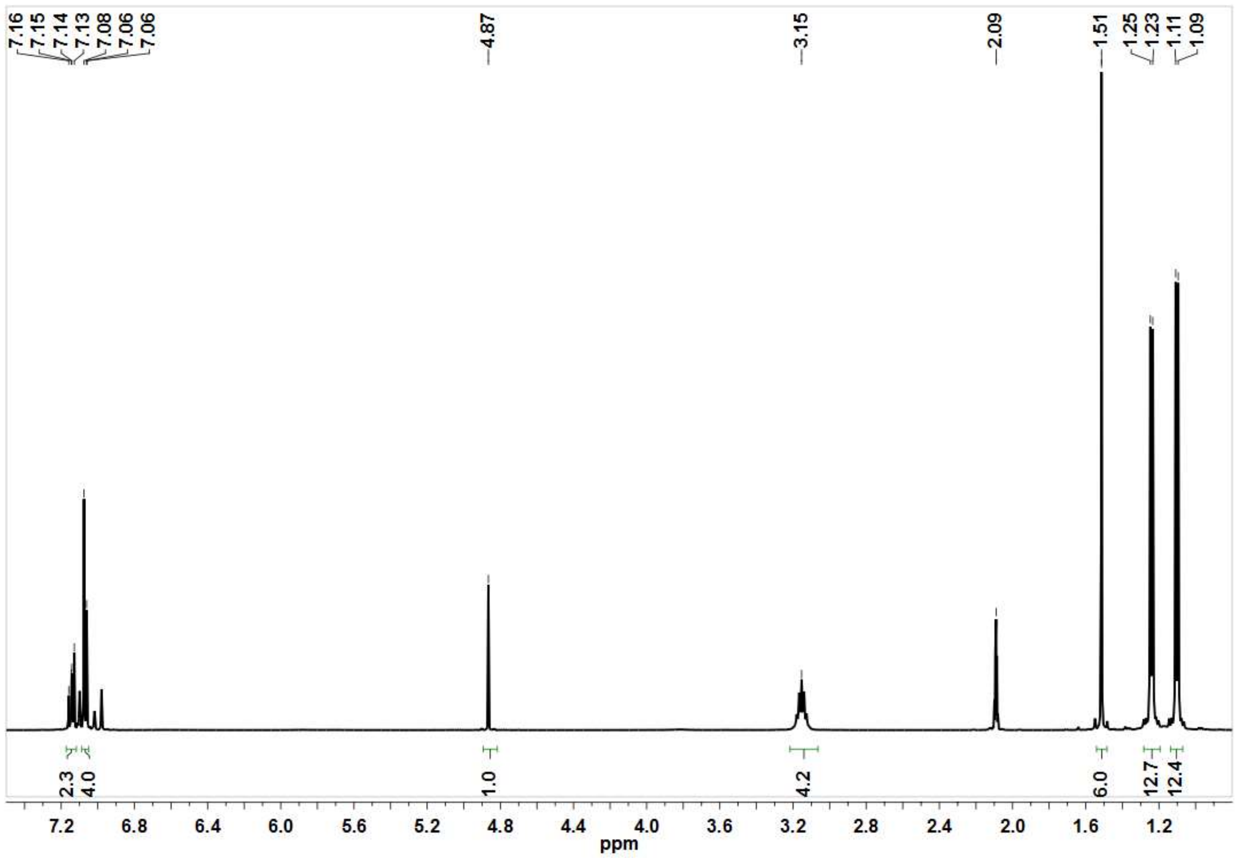

Supplementary Figure 17. ^{1}H NMR spectrum of LGaSbGa(Cl)L **5** in toluene-d_8 at room temperature.

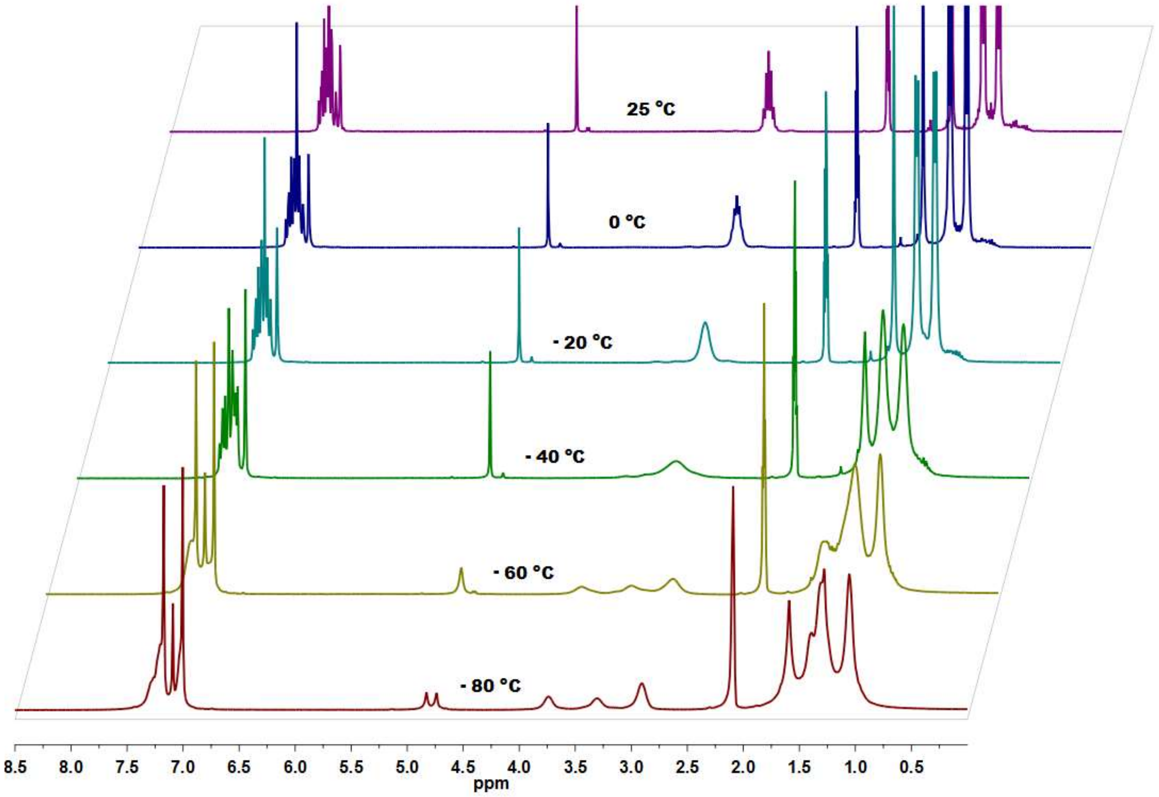

Supplementary Figure 18. Temperature-dependent ^{1}H NMR study of LGaSbGa(Cl)L **5** in toluene-d_8.

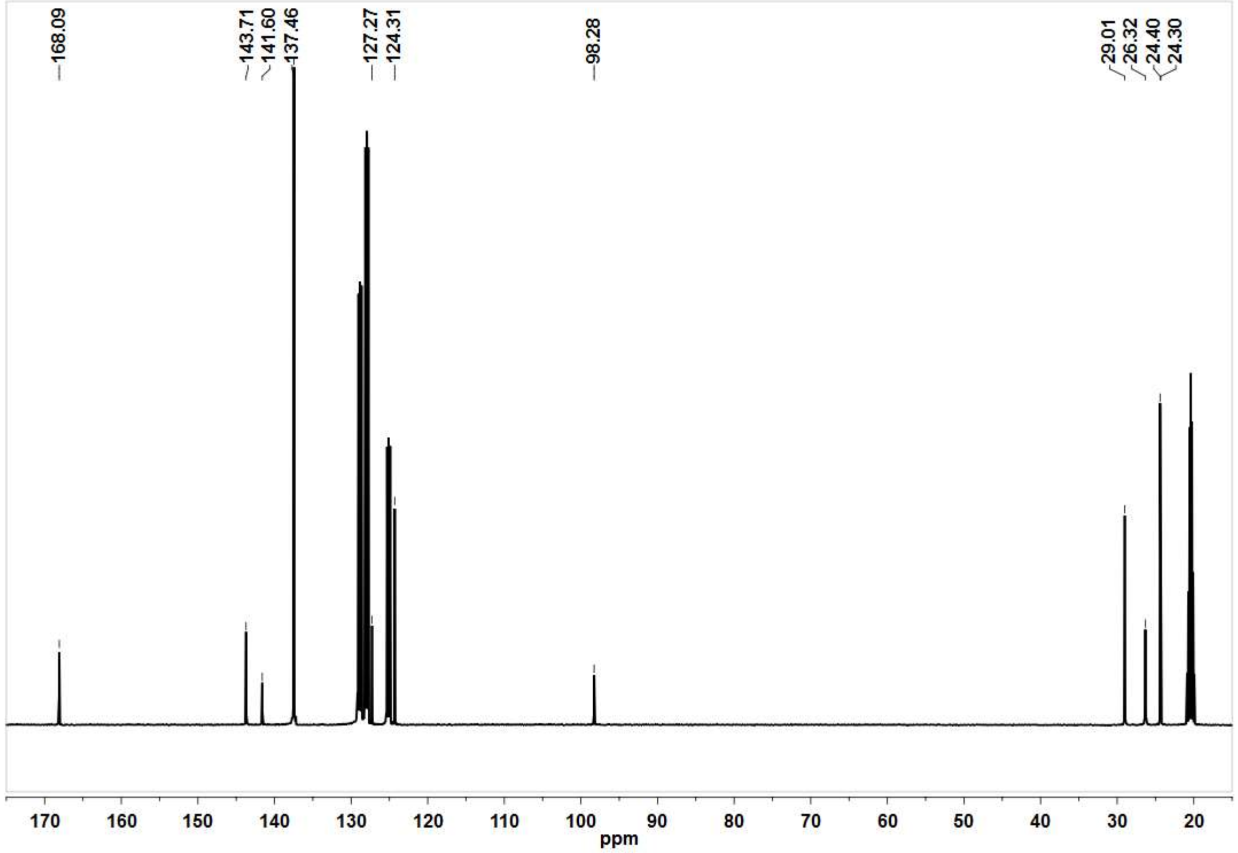

Supplementary Figure 19. ^{13}C NMR spectrum of LGaSbGa(Cl)L **5** in toluene-d_8 at room temperature.

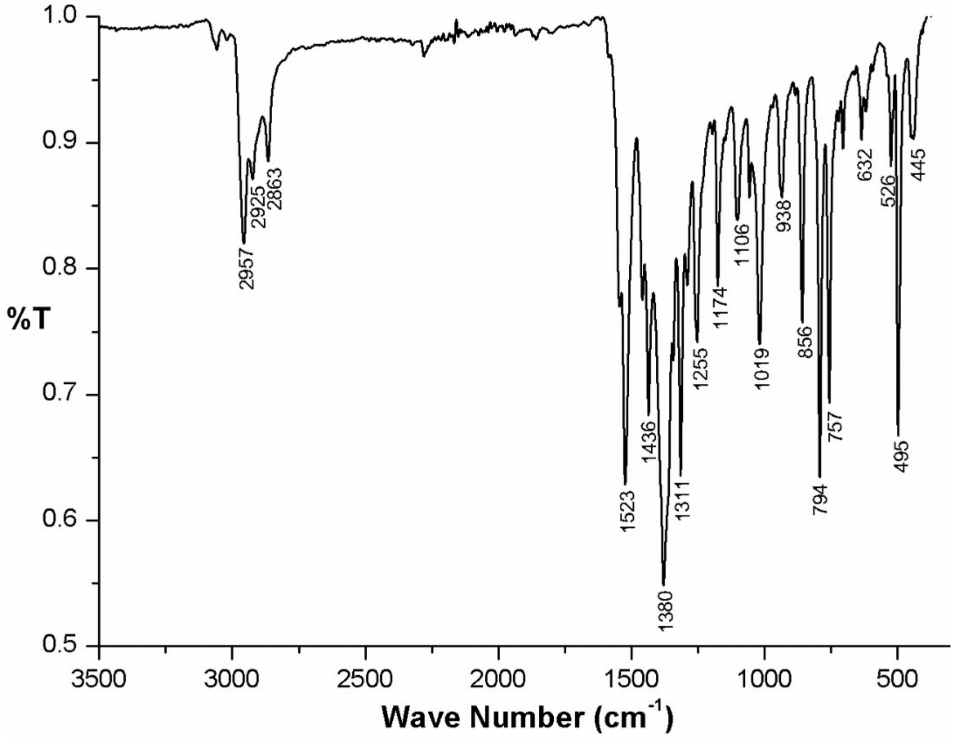

Supplementary Figure 20. ATR-IR spectrum of LGaSbGa(Cl)L **5**.

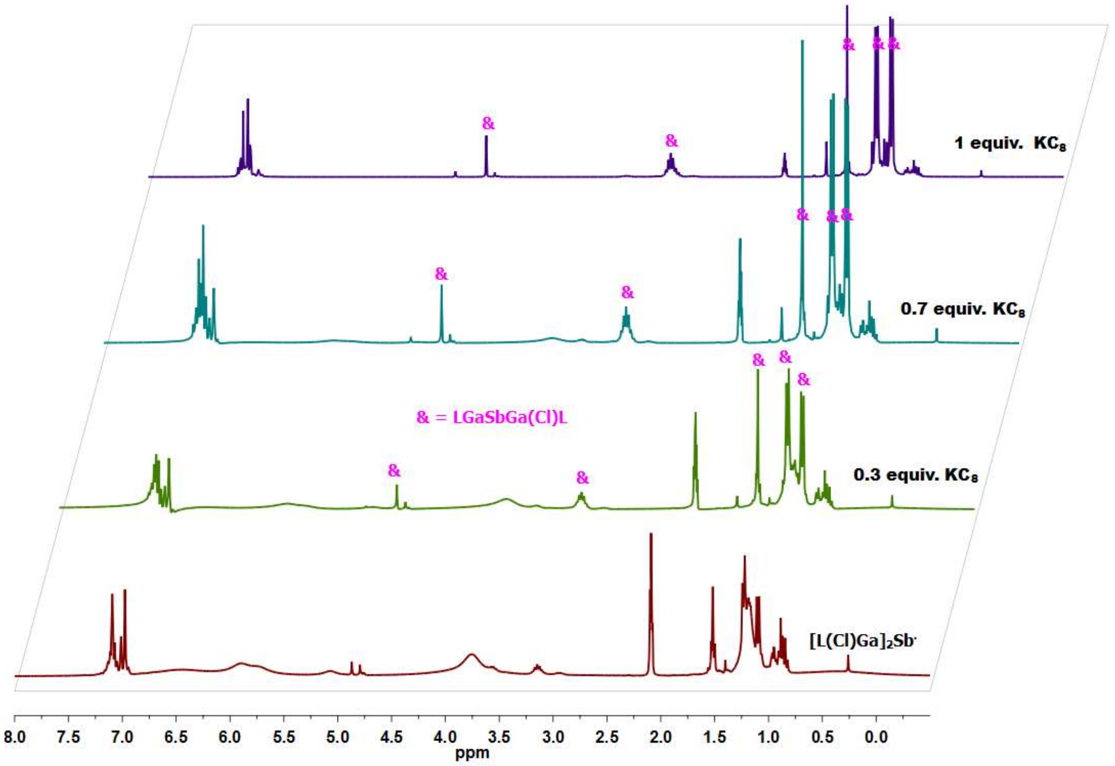

Supplementary Figure 21. The formation of LGaSbGa(Cl)L **5** from the reaction of $[L(Cl)Ga]_2Sb\cdot$ **1** with KC_8. ^1H NMR spectra were measured after adding 0.3, 0.7 and 1 equivalents of KC_8 in toluene-d_8 at room temperature.

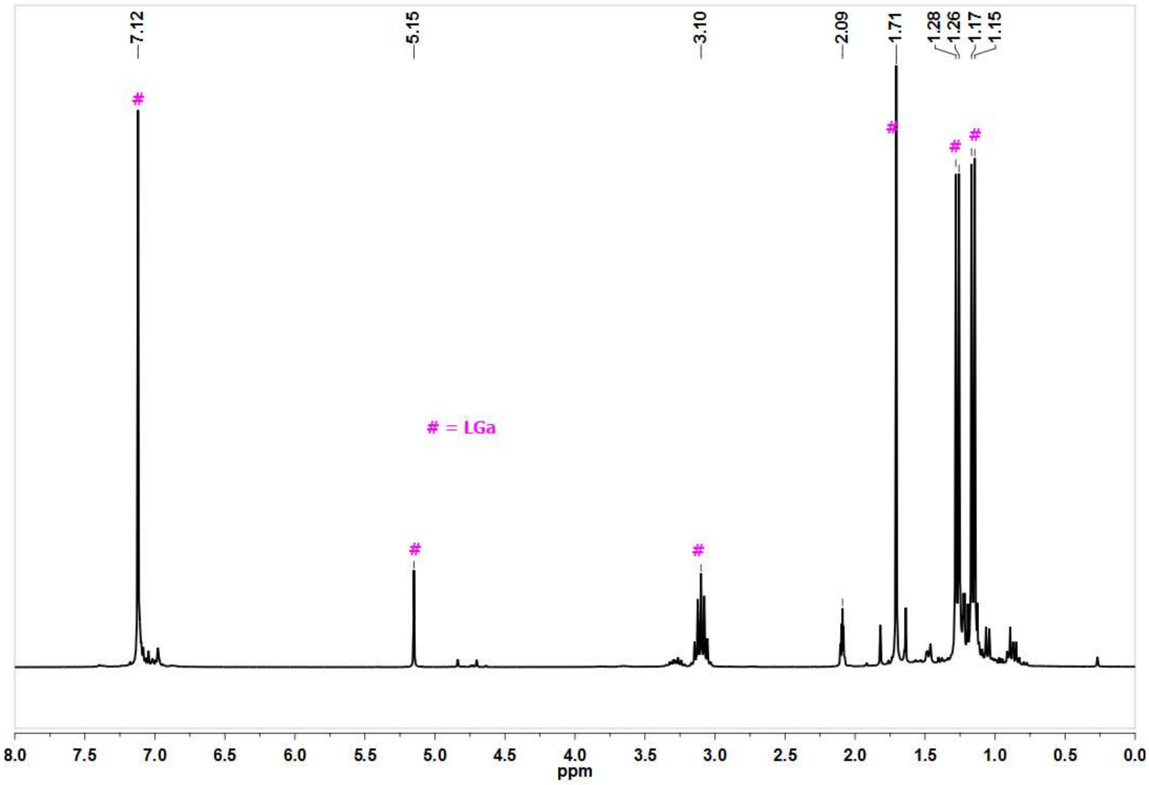

Supplementary Figure 22. The formation of LGa from the reaction of $[L(Cl)Ga]_2Sb\cdot$ **1** with two equivalents of KC_8 in toluene-d_8. The reaction mixture was stirred at room temperature for 1 day.

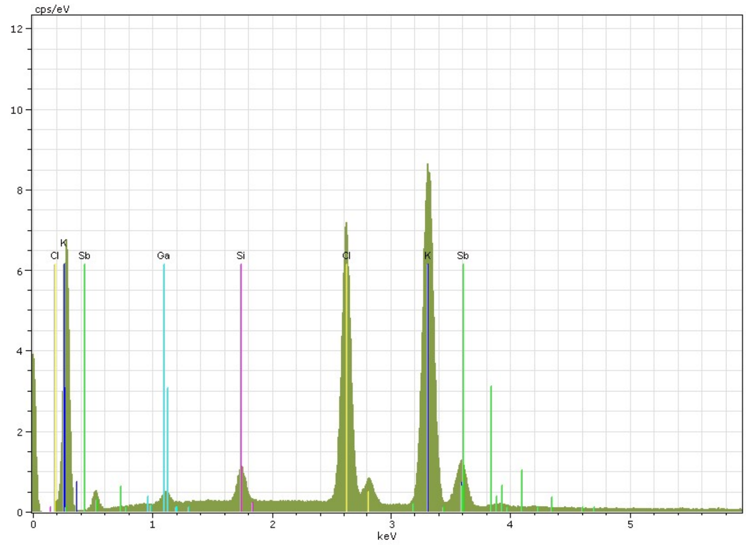

Supplementary Figure 23. EDX spectrum of the precipitate formed from the reaction of $[L(Cl)Ga]_2Sb\cdot$ **1** with two equivalents of KC_8 in toluene-d_8 showing the presence of Sb metal along with KCl.

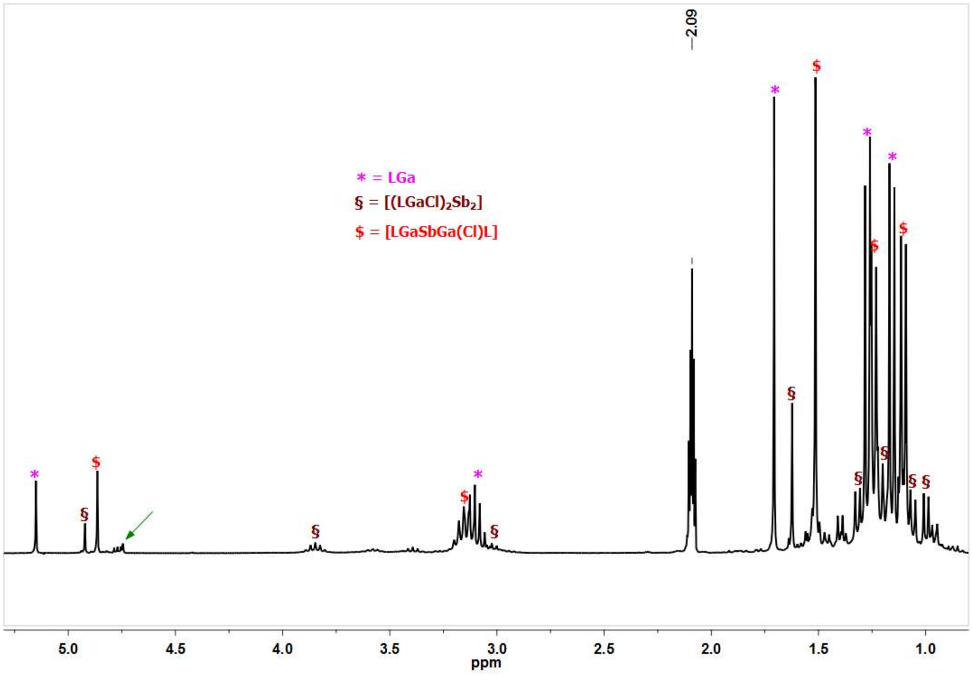

Supplementary Figure 24. Thermal decomposition study of LGaSbGa(Cl)L **5** in toluene-d_8. ^{1}H NMR spectrum of LGa=SbGa(Cl)L **5** in toluene-d_8 after heating at 120 °C for 30 h, showing the formation of $[(LGaCl)_2Sb_2]$ and LGa. The green arrow shows the formation of $[(LGaCl)_2Sb_4]$ and $LGaCl_2$ from $[(LGaCl)_2Sb_2]$ at 120 °C.

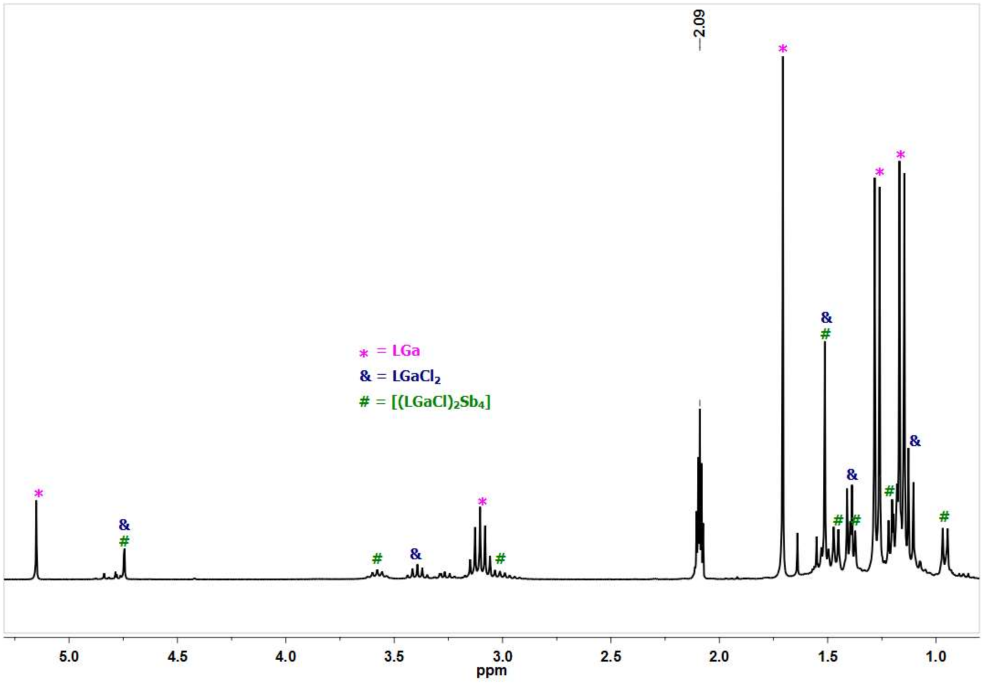

Supplementary Figure 25. Thermal decomposition study of LGaSbGa(Cl)L **5** in toluene-d_8. ^{1}H NMR spectrum of LGaSbGa(Cl)L **5** in toluene-d_8 after heating at 130 °C for 6 days, showing the formation of $[(LGaCl)_2Sb_4]$, $LGaCl_2$ and LGa.

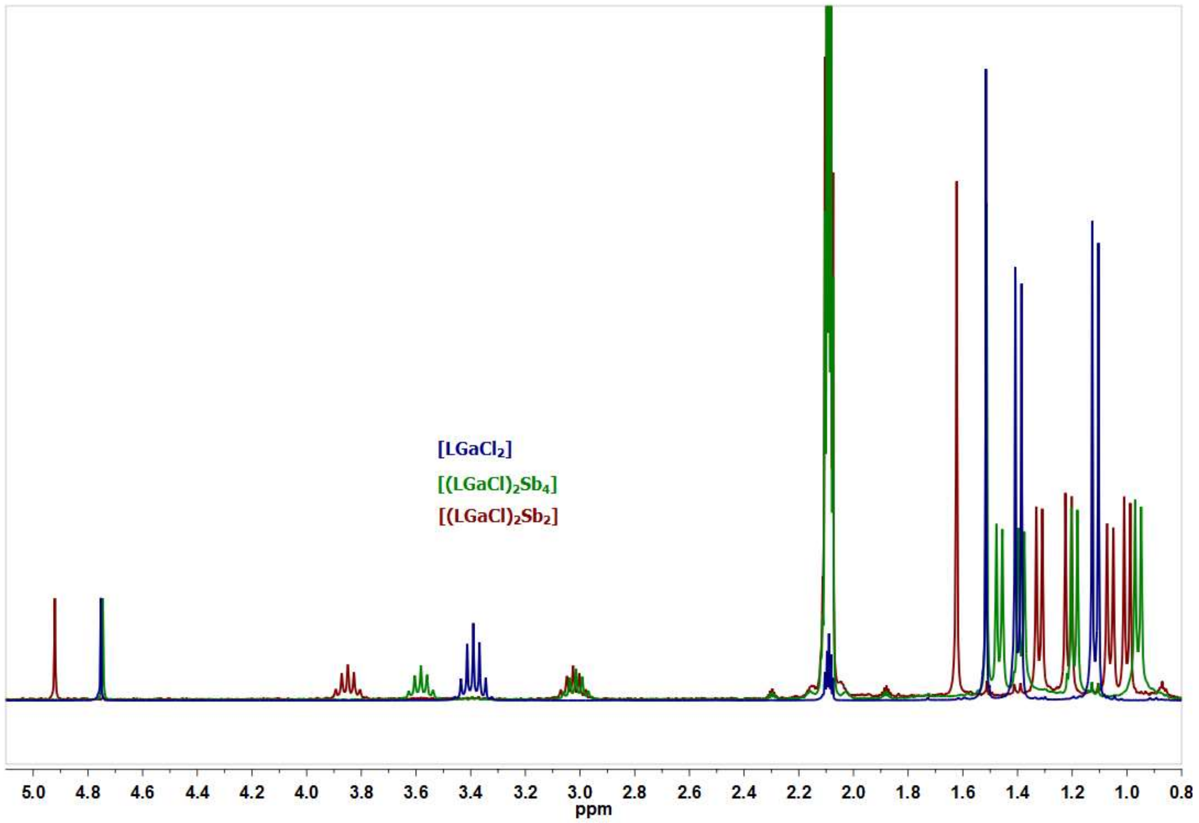

Supplementary Figure 26. A stacked ^{1}H NMR spectral plot of $LGaCl_2$, $[(LGaCl)_2Sb_4]$ and $[(LGaCl)_2Sb_2]$ in toluene-d_8. An overlapping view is presented here for comparing the spectra of thermal decomposition of LGaSbGa(Cl)L **5** at different conditions.

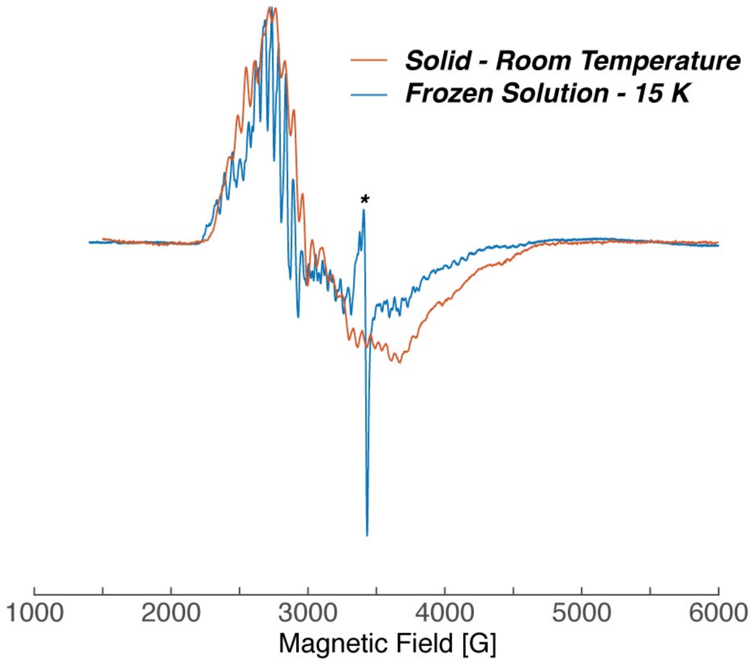

Supplementary Figure 27. X-band CW EPR spectra of **1** as a solid and frozen solution; * represents a small organic radical impurity in the frozen solution sample (not in the solid); impurity spin < 2%.

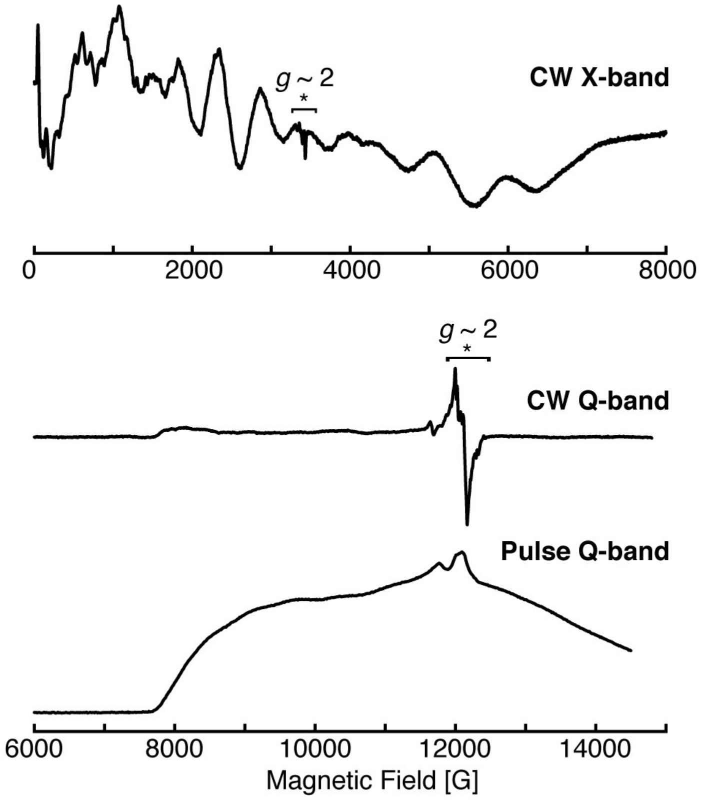

Supplementary Figure 28. CW X-band (top), CW- (middle) and pulse Q-band (bottom) EPR spectra of **4**, all collected at 10 K (CW) or 5 K (pulse). Parameters are described in the methods.

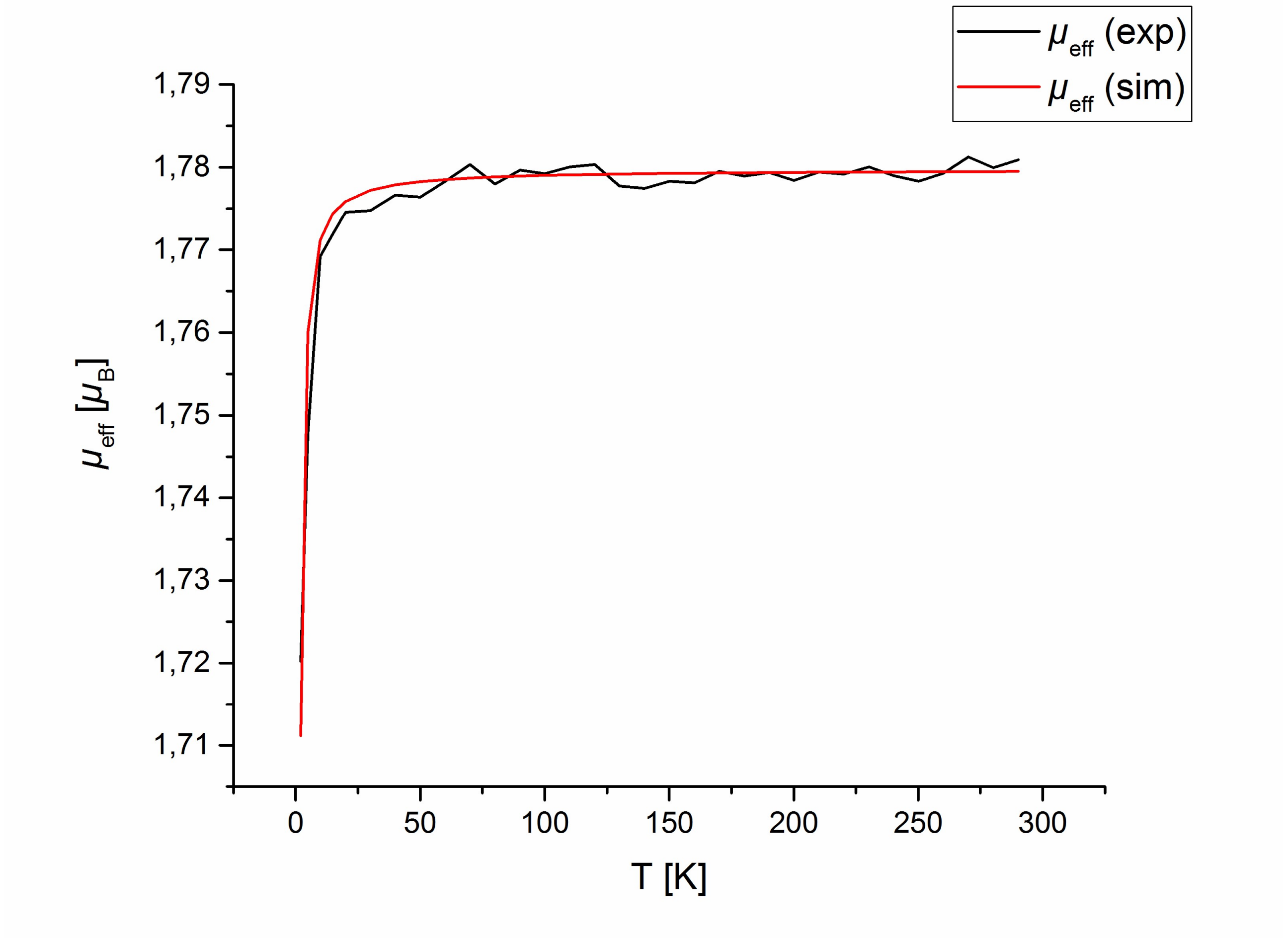

Supplementary Figure 29. Temperature dependent magnetic susceptibility data of solid (**4**). The red lines are spin Hamiltonian simulations obtained with average g values of g_{av} = 2.05. The decline of μ_{eff} below 10 K is due to usual field saturation, but weak antiferromagnetic intermolecular interaction was also taken into account, according to a molecular field correction *2zJ* = -2.9 K.

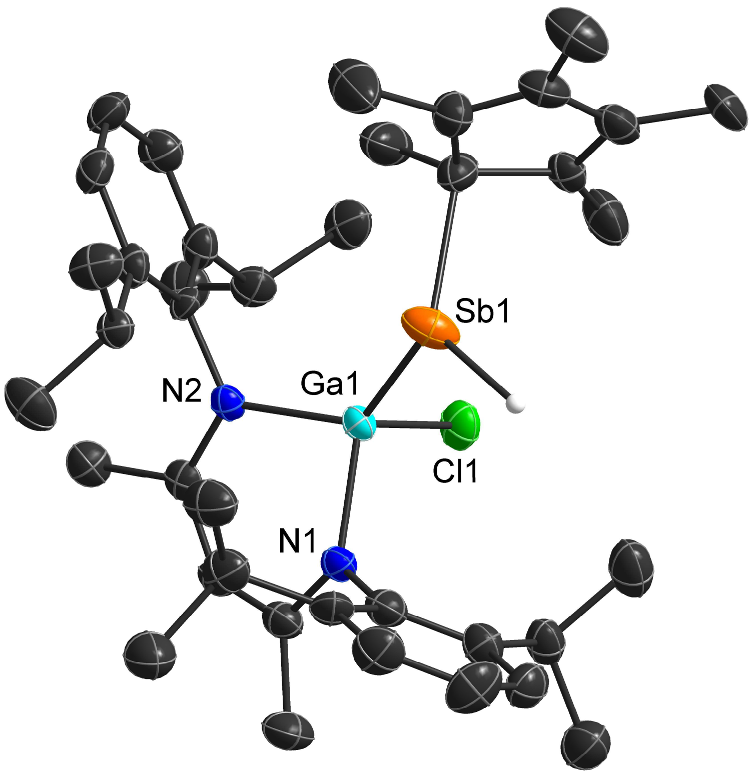

Supplementary Figure 30. Solid state structure of **2**.H-atoms were omitted for clarity except for Sb-H, displacement ellipsoids are drawn at the 50% probability level.

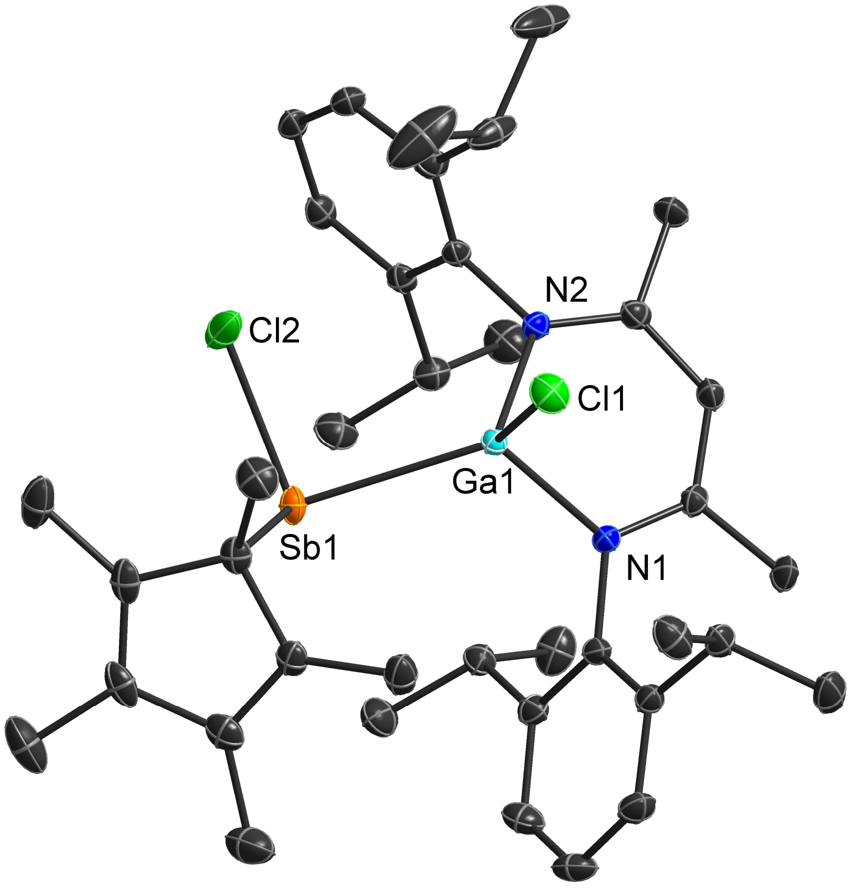

Supplementary Figure 31. Solid state structure of **3**. H-atoms were omitted for clarity, displacement ellipsoids are drawn at the 50% probability level.

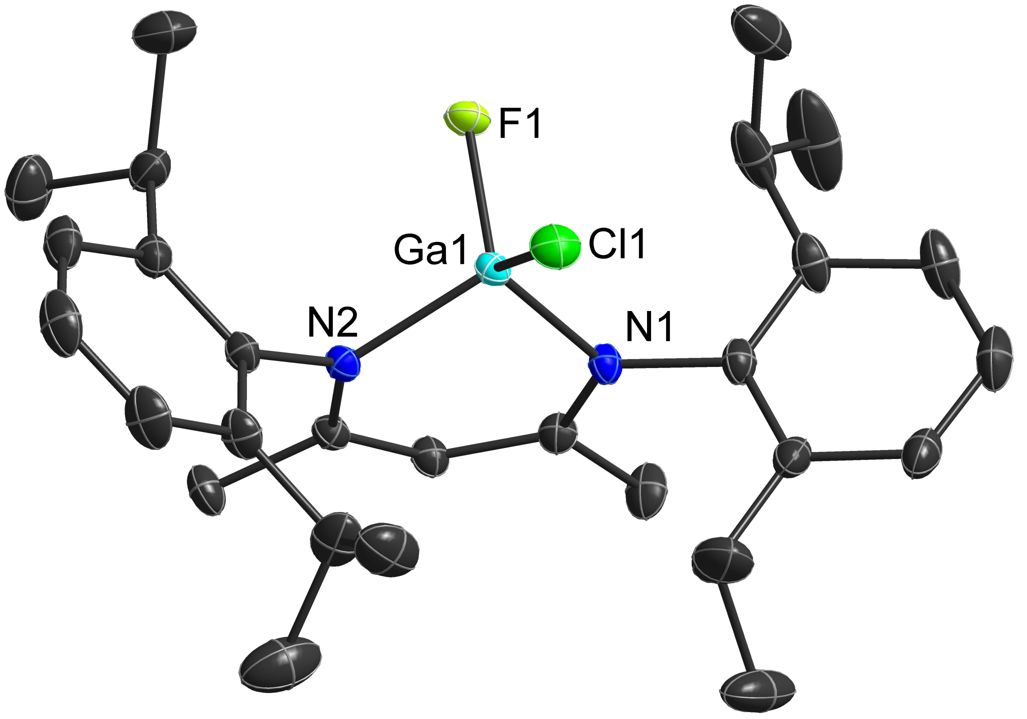

Supplementary Figure 32. Solid state structure of LGaClF. H-atoms and disordered atoms were omitted for clarity. Displacement ellipsoids are drawn at the 50% probability level.

Crystallographic details

Supplementary Table 1. Single Crystal X-ray diffraction data of **1 - 5** and LGaClF.

	**1**	**2**	**3**	**4**	**5**	**LGaClF**
Empirical formula	$C_{61}H_{89}Cl_2Ga_2N_4Sb$	$C_{39}H_{57}ClGaN_2Sb$	$C_{39}H_{56}Cl_2GaN_2Sb$	$C_{72}H_{98}BiGa_2I_2N_4$	$C_{67}H_{91}ClGa_2N_4Sb$	$C_{29}H_{41}ClFGaN_2$
M	1210.45	780.80	815.22	1621.77	1249.07	541.81
Crystal size [mm]	0.312 × 0.287 × 0.285	0.16 × 0.06 × 0.01	0.400 × 0.400 × 0.200	0.41 × 0.20 × 0.08	0.196 × 0.147 × 0.081	0.288 × 0.233 × 0.166
T [K]	100(2)	173(2)	100(2)	213(2)	100(2)	100(2)
Crystal system	monoclinic	triclinic	monoclinic	monoclinic	monoclinic	monoclinic
Space group	$P2_1/n$	P -1	$P2_1/n$	$P2_1/c$	$P2_1/c$	$P2_1/c$
a [Å]	12.2735(10)	10.1687(6)	14.0483(3)	14.4381(2)	16.0191(6)	8.9733(2)
b [Å]	41.270(3)	11.7017(6)	19.8994(4)	20.2337(4)	14.3320(5)	9.6029(3)
c [Å]	13.0798(11)	17.2183(9)	14.0749(3)	25.1401(4)	27.2963(10)	33.1607(8)
α [°]	90	88.906(4)	90	90	90	90
β [°]	111.682(4)	85.215(4)	102.556(2)	99.3540(10)	94.386(2)	92.1220(10)
γ [°]	90	73.128(4)	90	90	90	90
V [Å^3]	6156.5(9)	1953.77(19)	3840.59(14)	7246.7(2)	6248.5(4)	2855.49(13)
Z	4	2	4	4	4	4
D_{calc} [g·cm^{-3}]	1.306	1.327	1.410	1.486	1.328	1.260
μ(MoK_α [mm^{-1}])	1.430	1.476	1.573	4.053	1.370	1.083
Transmissions	0.75/0.66	0.641/0.585	1.00000/0.90304	0.835/0.456	0.75/0.65	0.75/0.68
F(000)	2520	808	1680	3236	2604	1144
Index ranges	$-20 \le h \le 20$	$-12 \le h \le 12$	$-19 \le h \le 20$	$-17 \le h \le 17$	$-24 \le h \le 23$	$-14 \le h \le 14$
	$-66 \le k \le 63$	$-14 \le k \le 14$	$-28 \le k \le 28$	$-24 \le k \le 24$	$-20 \le k \le 22$	$-12 \le k \le 15$
	$-21 \le l \le 21$	$-17 \le l \le 21$	$-19 \le l \le 20$	$-30 \le l \le 27$	$-41 \le l \le 39$	$-54 \le l \le 54$
θ_{max} [°]	36.631	26.000	31.163	25.799	33.330	36.433
Reflections collected	297653	17042	126863	103643	200068	76456
Independent reflections	29571	7686	11634	13956	23371	13387
R_{int}	0.0308	0.1226	0.0489	0.1499	0.0358	0.0217
Refined parameters	653	415	421	752	696	336
R_1 [$I > 2\sigma(I)$]	0.0589	0.0932	0.0265	0.0778	0.0327	0.0482
wR_2 [all data]	0.1313	0.1509	0.0616	0.1345	0.0853	0.1086
GooF	1.211	1.424	1.053	1.369	1.063	1.301
$\Delta\rho_{final}$ (max/min) [e·Å^{-3}]	2.011/-2.944	1.204/-1.235	0.841/-0.929	1.850/-0.930	1.143/-0.681	0.709/-1.247

Results from quantum chemical calculations for 1', 4' and 5'

Supplementary Table 2. Optimized Coordinates for 1'.

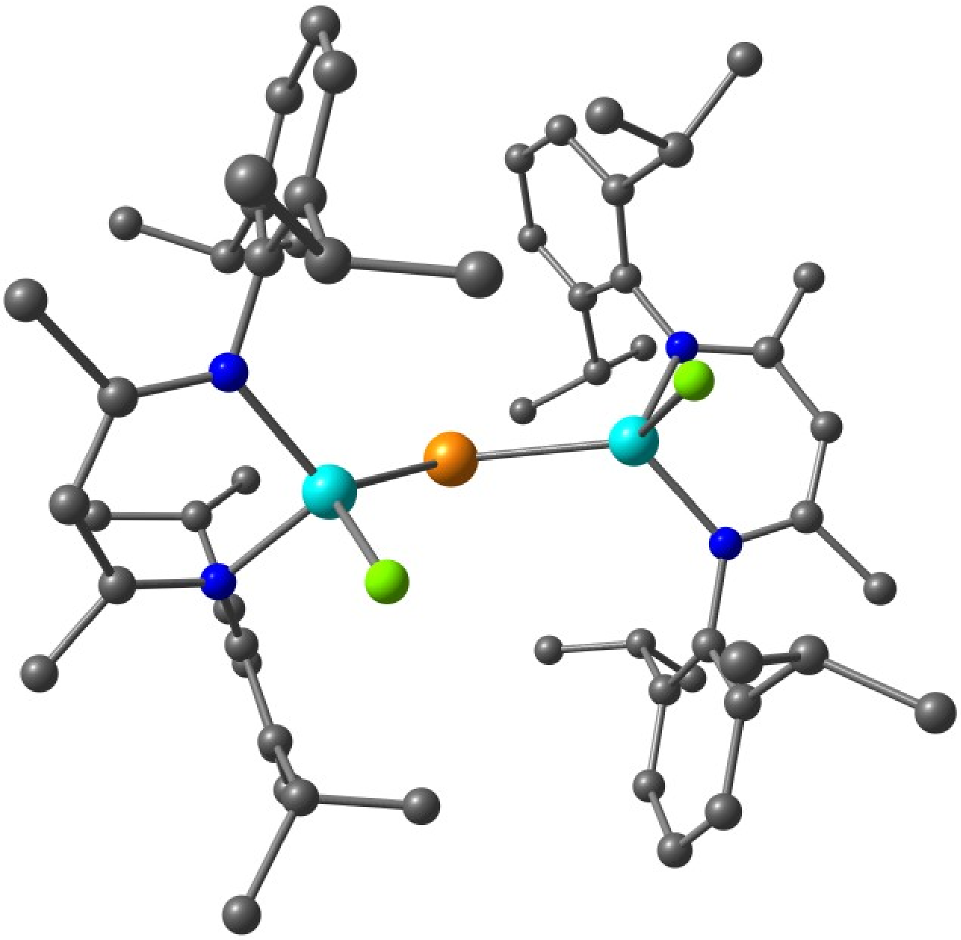

Ga	3.743876	4.782075	1.488566
Ga	0.458266	6.988239	-0.387480
Cl	5.102720	6.056091	0.185048
Cl	1.622602	8.930734	-0.105184
N	4.419986	5.115646	3.346475
N	4.500856	2.931005	1.266473
N	0.137785	6.984917	-2.362780
N	-1.421834	7.447732	0.135498
C	5.505575	4.440918	3.747043
C	6.093583	3.374685	3.037746
H	7.010429	2.987842	3.477010
C	5.604331	2.622060	1.950242
C	6.154408	4.786917	5.069048
H	7.048870	4.174420	5.235035
H	5.454238	4.606486	5.897812
H	6.436854	5.847370	5.116675
C	6.423798	1.406937	1.575139
H	5.820916	0.610081	1.123412
H	6.957400	1.007641	2.448197
H	7.181956	1.708412	0.832735
C	3.939120	1.996944	0.311036
C	4.355154	2.008061	-1.046702
C	3.737474	1.119830	-1.942440
H	4.051722	1.118334	-2.989110
C	2.742146	0.237889	-1.530477
H	2.269494	-0.436622	-2.249233
C	2.366799	0.211635	-0.189105
H	1.606962	-0.502814	0.137941

C	2.957475	1.066563	0.754633
C	5.466100	2.905807	-1.589556
H	5.850776	3.516327	-0.761813
C	4.931276	3.868159	-2.663269
H	4.551372	3.319615	-3.539627
H	5.731392	4.542143	-3.010445
H	4.114178	4.493454	-2.278487
C	6.642803	2.082215	-2.149814
H	6.332864	1.464835	-3.008873
H	7.070606	1.405327	-1.394000
H	7.446489	2.752343	-2.496211
C	2.592291	0.864467	2.226625
H	3.052850	1.674640	2.811077
C	1.076936	0.902267	2.487336
H	0.636547	1.859351	2.167916
H	0.868883	0.765996	3.561557
H	0.551209	0.096743	1.951519
C	3.179096	-0.470859	2.735370
H	2.729115	-1.321740	2.197362
H	2.968621	-0.604646	3.809824
H	4.269732	-0.525114	2.590129
C	-0.836007	7.764782	-2.844615
C	-1.795901	8.424622	-2.047919
H	-2.466073	9.085607	-2.594986
C	-2.135119	8.215544	-0.697384
C	-0.978464	7.951331	-4.339179
H	-0.047634	8.319179	-4.790942
H	-1.784881	8.658596	-4.568303
H	-1.209184	6.992875	-4.828969
C	-3.388010	8.905241	-0.201470
H	-3.926391	9.388825	-1.026382
H	-3.125770	9.673296	0.541529
H	-4.065415	8.203028	0.303442
C	0.952228	6.227090	-3.290880
C	2.081972	6.823659	-3.912060
C	2.788046	6.076576	-4.868170
H	3.657213	6.524560	-5.355834
C	2.407847	4.780565	-5.208497
H	2.970125	4.221294	-5.961143
C	1.323087	4.192463	-4.561325
H	1.037960	3.169371	-4.816276
C	0.583385	4.889494	-3.592655
C	2.594490	8.222405	-3.569941
H	1.878007	8.693094	-2.883206
C	3.940942	8.127117	-2.826606
H	4.719124	7.695011	-3.478489
H	3.866813	7.499521	-1.928033
H	4.281095	9.127687	-2.511261
C	2.732260	9.136830	-4.799904
H	3.479940	8.755382	-5.514634
H	3.066859	10.138532	-4.481755
H	1.779743	9.255082	-5.340799
C	-0.592711	4.178623	-2.921560
H	-0.925170	4.802777	-2.078624
C	-1.798501	4.029111	-3.869024
H	-1.543756	3.405509	-4.741993
H	-2.154348	5.002353	-4.242698
H	-2.639300	3.542248	-3.346459
C	-0.182917	2.808779	-2.348149
H	0.129639	2.107636	-3.138704
H	-1.030855	2.349115	-1.814649
H	0.655279	2.897789	-1.639009
C	-1.976561	7.104347	1.427708

C	-1.786771	7.965238	2.537708
C	-2.376695	7.619690	3.764753
H	-2.242733	8.280345	4.625600
C	-3.124514	6.453508	3.907626
H	-3.577691	6.198424	4.869566
C	-3.285874	5.604102	2.814073
H	-3.865661	4.687546	2.935841
C	-2.724202	5.901997	1.562809
C	-0.934968	9.229245	2.468534
H	-0.661108	9.400164	1.419299
C	0.375117	9.020709	3.247448
H	0.961063	8.188488	2.831780
H	0.180369	8.792722	4.308094
H	1.006805	9.922252	3.199061
C	-1.671091	10.491108	2.954950
H	-1.039823	11.379768	2.785420
H	-1.903842	10.450370	4.031779
H	-2.619213	10.647842	2.416835
C	-2.967513	4.942870	0.396243
H	-2.192570	5.144494	-0.358514
C	-2.846225	3.463310	0.800441
H	-2.945051	2.821402	-0.089460
H	-3.640364	3.162284	1.501872
H	-1.874655	3.249166	1.273059
C	-4.335260	5.184947	-0.275536
H	-4.438132	6.211024	-0.658753
H	-5.157196	5.002532	0.437282
H	-4.469044	4.498212	-1.126925
C	3.857229	6.135764	4.213221
C	4.335066	7.473503	4.142924
C	3.767386	8.433219	4.996113
H	4.130014	9.463026	4.949756
C	2.764858	8.104474	5.904872
H	2.337611	8.870147	6.557689
C	2.313414	6.789421	5.974389
H	1.535096	6.529040	6.696079
C	2.843480	5.785863	5.146637
C	5.456962	7.931183	3.210221
H	5.810432	7.058651	2.645936
C	6.661747	8.498937	3.987147
H	7.472600	8.763104	3.289116
H	6.395424	9.410987	4.546363
C	4.945830	8.959438	2.184929
H	5.749002	9.230489	1.479831
H	4.607271	9.884866	2.680277
H	4.108199	8.559616	1.596937
C	2.325596	4.361329	5.345151
H	2.825663	3.707742	4.614973
C	0.809408	4.261479	5.103947
H	0.534712	4.591784	4.089690
H	0.245578	4.882704	5.817003
H	0.468033	3.221060	5.233871
C	2.667629	3.837729	6.756007
H	2.328553	2.795167	6.875575
H	2.166744	4.438712	7.531999
H	3.749878	3.869603	6.957131
Sb	1.156930	4.766193	0.893354
H	7.067229	7.775061	4.711850

Supplementary Table 3. Optimized Coordinates for 4'

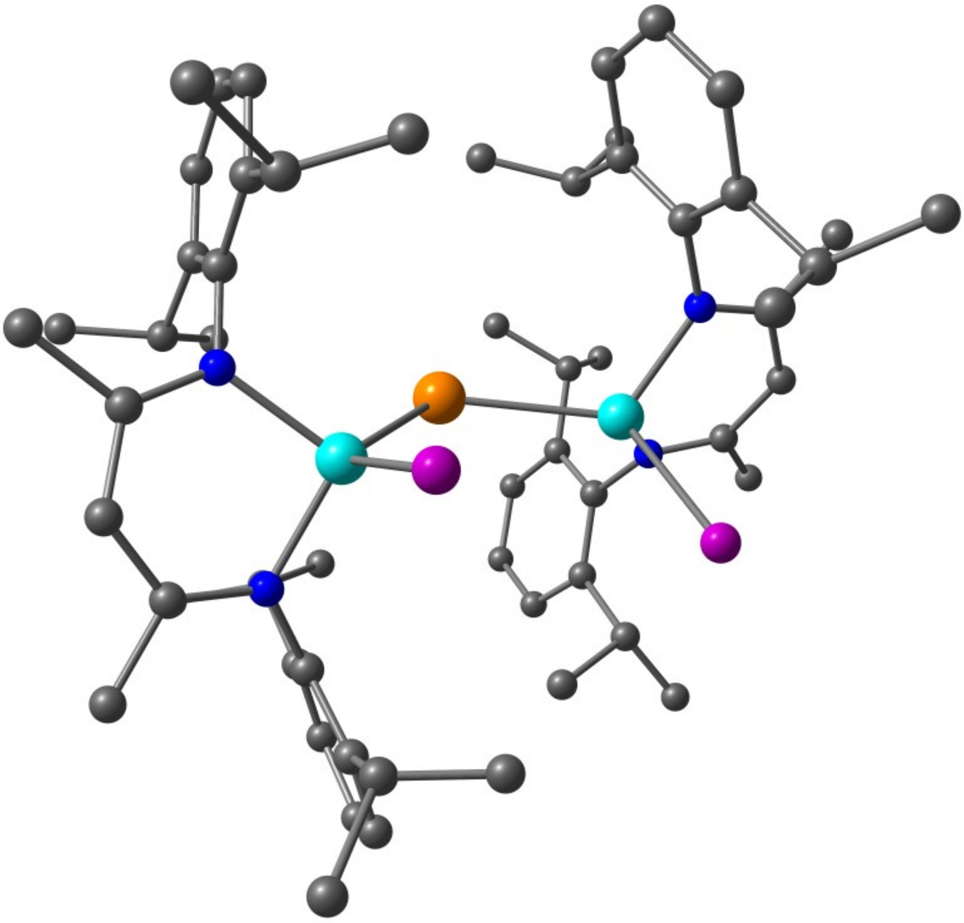

Bi	0.783655	14.304126	13.837753
I	1.785035	15.720176	18.229708
I	0.508339	18.92917	14.740982
Ga	2.299637	14.140588	16.123696
Ga	-0.429766	16.742211	13.506139
N	2.158893	12.268417	16.838848
N	4.303636	14.195509	15.990059
N	-0.386717	17.272466	11.569862
N	-2.41139	16.891121	13.753238
C	3.174178	11.797092	17.573287
C	4.44875	12.390584	17.620001
H	5.162621	11.887646	18.269605
C	5.008116	13.387784	16.791684
C	2.988419	10.545619	18.400862
H	3.94363	10.203092	18.817678
H	2.541795	9.729485	17.817474
H	2.302125	10.750955	19.236224
C	6.518671	13.469792	16.811548
H	6.935521	12.737267	17.511919
H	6.873098	14.467866	17.09909
H	6.930229	13.265276	15.811595
C	1.002651	11.42377	16.610494
C	1.020866	10.52103	15.509745
C	-0.102364	9.707093	15.295246
H	-0.104823	9.010225	14.453702
C	-1.216202	9.763955	16.131735
H	-2.081246	9.123534	15.937476
C	-1.21331	10.637599	17.216909
H	-2.08197	10.670121	17.880693
C	-0.116813	11.475249	17.482398
C	2.229664	10.347584	14.584665

H	2.93679	11.160705	14.801778
C	2.966337	9.016538	14.847621
H	3.865846	8.947303	14.214899
H	2.319114	8.156998	14.605017
H	3.287614	8.914192	15.895428
C	1.856161	10.439809	13.094658
H	2.758906	10.349539	12.469381
H	1.370194	11.398229	12.855319
H	1.169335	9.632902	12.794356
C	-0.175123	12.375959	18.713107
H	0.764994	12.94173	18.759699
C	-0.305704	11.578449	20.02641
H	-0.234012	12.261326	20.889765
H	0.479936	10.814967	20.138238
H	-1.278301	11.062872	20.089925
C	-1.318713	13.400165	18.612092
H	-1.263654	14.119981	19.445075
H	-2.303325	12.90607	18.658395
H	-1.272007	13.973738	17.674982
C	5.043048	15.071302	15.096941
C	5.296276	14.642871	13.766838
C	6.152785	15.411863	12.963304
H	6.378313	15.07552	11.948976
C	6.731614	16.586774	13.435146
H	7.406153	17.164934	12.797519
C	6.421367	17.032205	14.717085
H	6.850842	17.970502	15.075057
C	5.580078	16.29931	15.569627
C	4.699801	13.370822	13.166621
H	3.898976	13.024872	13.837759
C	5.735013	12.234031	13.074991
H	5.275362	11.325567	12.65182
H	6.15036	11.970994	14.060368
H	6.576227	12.518561	12.421962
C	4.064716	13.64097	11.789291
H	3.556546	12.737934	11.415242
H	4.817841	13.92982	11.03944
H	3.321508	14.452292	11.837276
C	5.282255	16.890842	16.947958
H	4.695305	16.15825	17.519406
C	6.552968	17.21176	17.757594
H	6.278508	17.592873	18.755037
H	7.160689	17.989502	17.267251
H	7.193451	16.327381	17.901435
C	4.418887	18.159747	16.805266
H	4.136086	18.550615	17.796654
H	3.492386	17.957606	16.24767
H	4.970455	18.952928	16.273187
C	-3.06464	17.761278	12.976826
C	-2.47881	18.455821	11.893715
H	-3.118644	19.209472	11.437945
C	-1.303444	18.165029	11.173949
C	-4.535402	18.023685	13.207684
H	-4.90914	18.781105	12.508909
H	-4.734511	18.362574	14.232534
H	-5.118478	17.102003	13.062095
C	-1.1336	18.877246	9.851274
H	-1.827285	19.724008	9.7747
H	-1.337783	18.193777	9.013798
H	-0.105945	19.243218	9.721495
C	-3.143816	16.141656	14.758275
C	-3.589512	14.826232	14.455083
C	-4.355135	14.137122	15.408991

H	-4.718774	13.133726	15.179211
C	-4.666798	14.7052	16.641154
H	-5.265739	14.15279	17.370343
C	-4.196663	15.980189	16.940095
H	-4.430149	16.41856	17.913183
C	-3.433179	16.721262	16.024048
C	-3.307079	14.127035	13.126386
H	-2.493912	14.673055	12.624754
C	-4.530773	14.167045	12.190062
H	-4.304279	13.658534	11.238235
H	-4.840319	15.196405	11.953815
H	-5.391816	13.652571	12.648271
C	-2.838664	12.672681	13.3283
H	-2.509312	12.238559	12.372472
H	-3.645886	12.029996	13.714665
H	-1.997252	12.610649	14.036024
C	-2.948299	18.099553	16.472418
H	-2.435047	18.583004	15.629174
C	-4.101945	19.019834	16.915912
H	-3.708388	20.007747	17.205623
H	-4.628819	18.610389	17.792989
H	-4.848939	19.174213	16.120883
C	-1.922955	17.959512	17.613516
H	-1.515265	18.945557	17.891195
H	-1.079935	17.31523	17.325726
H	-2.389914	17.52237	18.51189
C	0.585631	16.788752	10.606638
C	0.210848	15.743852	9.715348
C	1.157332	15.273345	8.790867
H	0.878534	14.470338	8.10406
C	2.438916	15.813403	8.719853
H	3.161082	15.431305	7.993368
C	2.787868	16.84953	9.581541
H	3.787987	17.284723	9.515607
C	1.884066	17.358924	10.528715
C	-1.181305	15.111951	9.679249
H	-1.803047	15.605514	10.440502
C	-1.131559	13.612415	10.020157
H	-0.707161	13.441886	11.021643
H	-0.513244	13.053796	9.299971
H	-2.141844	13.174056	9.996631
C	-1.864483	15.319104	8.311656
H	-2.881941	14.896217	8.322847
H	-1.305392	14.812835	7.508298
H	-1.941981	16.384546	8.0443
C	2.353139	18.518476	11.403337
H	1.513722	18.828739	12.038502
C	3.499061	18.084385	12.333147
H	3.816218	18.921208	12.97591
H	4.377207	17.748482	11.75947
H	3.200511	17.257601	12.995307
C	2.771892	19.74543	10.569875
H	3.058096	20.575387	11.236895
H	1.953646	20.10006	9.922869
H	3.638426	19.526886	9.924668

Supplementary Table 4. Optimized Coordinates for 5'

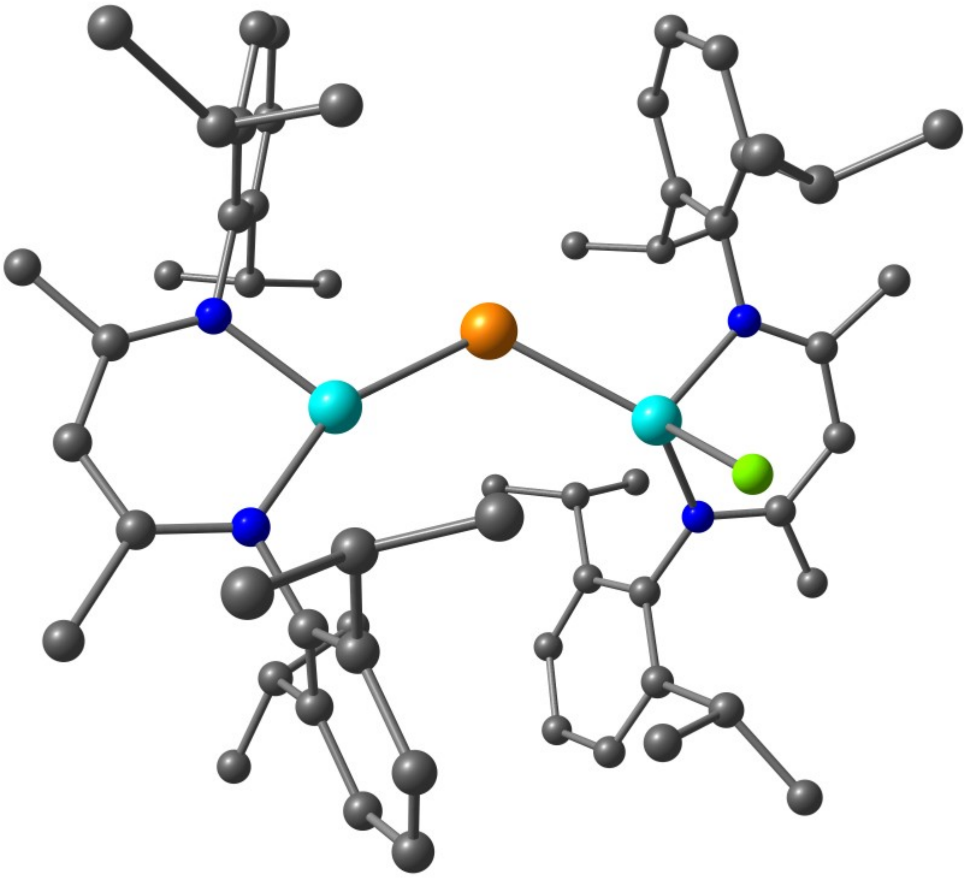

Sb	4.330085	5.501028	9.577815
Ga	1.963474	6.638610	9.836708
Ga	4.925695	4.397012	7.375526
Cl	1.295335	8.224706	8.278633
N	0.167504	5.870097	10.363303
N	2.146736	7.784689	11.511784
N	4.513634	3.766220	5.552704
N	6.809566	3.725781	7.387174
C	-0.635601	6.583925	11.165319
C	-0.242863	7.734776	11.869729
H	-1.032977	8.218233	12.443749
C	1.053472	8.227690	12.124378
C	-2.075236	6.156579	11.377051
H	-2.672260	6.337415	10.470967
H	-2.528255	6.721088	12.200762
H	-2.154029	5.084307	11.602097
C	1.119353	9.305941	13.186752
H	0.597728	10.202274	12.813716
H	2.139859	9.588550	13.461974
H	0.585703	8.979327	14.091528
C	-0.361658	4.646316	9.802032
C	-0.081160	3.410770	10.447091
C	-0.691800	2.248262	9.951266
H	-0.499524	1.291807	10.440323
C	-1.537349	2.284892	8.841892
H	-2.002430	1.367298	8.470584
C	-1.768903	3.496483	8.195341
H	-2.410333	3.518640	7.310157
C	-1.193955	4.692678	8.654168
C	0.845162	3.320982	11.662885
H	1.598236	4.116377	11.547830

C	1.614520	1.991682	11.738993
H	0.957518	1.150563	12.017553
H	2.399434	2.058003	12.509138
H	2.101658	1.747637	10.782348
C	0.109480	3.563126	12.996358
H	-0.311283	4.575891	13.067528
H	0.805503	3.429645	13.841226
H	-0.716650	2.844465	13.127255
C	-1.464123	5.976644	7.869427
H	-1.035790	6.814860	8.432914
C	-0.737416	5.924325	6.515515
H	-1.158956	5.135746	5.872653
H	0.335333	5.718238	6.638483
H	-0.833488	6.883863	5.981344
C	-2.961467	6.266723	7.657904
H	-3.087178	7.217395	7.112634
H	-3.509065	6.351928	8.609392
H	-3.453436	5.483364	7.058096
C	3.449926	8.154577	12.011351
C	4.027592	7.390218	13.059364
C	5.311155	7.738988	13.507939
H	5.771033	7.162382	14.314701
C	6.007011	8.813770	12.954992
H	7.001858	9.075431	13.326497
C	5.415073	9.568917	11.943763
H	5.954467	10.422307	11.524877
C	4.134436	9.264776	11.457868
C	3.280247	6.258162	13.766383
H	2.382484	6.023945	13.175142
C	4.105017	4.963415	13.862924
H	4.436125	4.626115	12.868583
H	3.499817	4.160124	14.315441
H	4.999569	5.092656	14.494177
C	2.809888	6.701550	15.169323
H	2.176168	7.599268	15.126237
H	3.670396	6.936039	15.818687
H	2.230475	5.899076	15.656175
C	3.508559	10.173584	10.403915
H	2.560797	9.719807	10.089791
C	4.381857	10.287876	9.142556
H	3.850964	10.858358	8.362690
H	4.617938	9.292771	8.734009
H	5.331566	10.810416	9.348309
C	3.183138	11.567248	10.983544
H	4.095316	12.094919	11.310370
H	2.511831	11.498707	11.853270
H	2.685437	12.193453	10.223426
C	5.359264	3.021942	4.815139
C	6.638066	2.633042	5.235237
H	7.194572	2.017386	4.530548
C	7.336370	2.989329	6.405670
C	4.934739	2.550901	3.439379
H	4.557260	3.383502	2.828842
H	4.113688	1.820877	3.506673
H	5.774587	2.076683	2.915667
C	8.777787	2.539159	6.481545
H	8.964428	1.724052	5.770801
H	9.059516	2.211282	7.489735
H	9.449747	3.372481	6.217832
C	3.207120	4.035894	4.994683
C	2.214691	3.023928	5.086259
C	0.985574	3.249718	4.452292
H	0.206996	2.484939	4.494983

C	0.737267	4.438314	3.762341
H	-0.226740	4.597110	3.272010
C	1.711620	5.431208	3.712185
H	1.498042	6.360304	3.178310
C	2.965104	5.258224	4.323618
C	2.465433	1.730232	5.865576
H	3.544335	1.519687	5.830812
C	2.096731	1.908352	7.350824
H	1.025210	2.131067	7.472934
H	2.652479	2.735743	7.819992
H	2.327121	0.991013	7.919605
C	1.768224	0.494490	5.273946
H	2.028221	0.348153	4.212370
H	0.670484	0.554347	5.350799
H	2.085717	-0.405854	5.824841
C	4.013276	6.367440	4.204836
H	4.861053	6.096843	4.853061
C	3.481437	7.728126	4.690437
H	2.653933	8.088292	4.057315
H	4.283753	8.482928	4.639488
H	3.115452	7.678416	5.727937
C	4.562835	6.495623	2.768354
H	3.772713	6.816829	2.068946
H	4.976583	5.545286	2.395914
H	5.369108	7.247235	2.728983
C	7.681629	4.184371	8.448070
C	7.754155	3.474394	9.674189
C	8.633839	3.951525	10.659829
H	8.714091	3.414438	11.607917
C	9.411107	5.090667	10.454639
H	10.089102	5.440673	11.238210
C	9.315443	5.783941	9.248272
H	9.918757	6.682286	9.097072
C	8.458062	5.351993	8.224982
C	6.952334	2.202167	9.960317
H	6.202499	2.088212	9.162520
C	7.843835	0.943995	9.950871
H	7.238643	0.049086	10.173603
H	8.631374	1.014293	10.719750
H	8.341052	0.783935	8.981699
C	6.186384	2.280743	11.294220
H	5.546649	3.174656	11.331466
H	6.872279	2.300179	12.157327
H	5.538761	1.395759	11.412311
C	8.379786	6.169080	6.931078
H	7.774526	5.604795	6.206810
C	9.758811	6.393064	6.281706
H	10.274479	5.440782	6.077907
H	10.417856	7.001990	6.921638
H	9.646265	6.925872	5.323123
C	7.666240	7.514913	7.170808
H	7.534633	8.058303	6.220222
H	8.253842	8.156783	7.848099
H	6.674186	7.367519	7.626622

Supplementary Methods

Synthetic methods.

All manipulations were performed in an atmosphere of purified argon using standard Schlenk and glove-box techniques. Toluene and hexane were dried using mBraun Solvent Purification System. Benzene was carefully dried over Na. Deuterated solvents were dried over activated molecular sieves (4 Å) and degassed prior to use. The anhydrous nature of the solvents was verified by Karl Fischer titration. LGa {L = $HC[C(Me)N(2,6\text{-}i\text{Pr}_2C_6H_3)]_2$},[1] Cp^*SbCl_2,[2] and Cp^*BiI_2[3] were prepared according to literature methods and other chemicals were obtained from commercial sources and purified prior to use. Microanalyses were performed at the elemental analysis laboratory of University of Duisburg-Essen. The melting points were measured using a Thermo Scientific 9300 apparatus.

Spectroscopic methods.

NMR Spectroscopy. The ^1H (300 and 500 MHz) and $^{13}C\{^1H\}$ (75.5 and 150 MHz) spectra were recorded using a Bruker Avance DPX-300 or Bruker Avance III HD spectrometer and the spectra were referenced to internal C_6D_5H (^1H: $\delta = 7.154$; ^{13}C: $\delta = 128.39$) and $C_6D_5CHD_2$ (^1H: $\delta = 2.09$; ^{13}C: $\delta = 20.40$).

IR Spectroscopy. IR spectra were recorded with an ALPHA-T FT-IR spectrometer equipped with a single reflection ATR sampling module. The IR spectrometer was placed in a glovebox to guarantee measurements under inert gas conditions.

EPR Spectroscopy. X-band (9.634 GHz) continuous-wave (CW) electron paramagnetic resonance (EPR) spectra were collected at 15 K on a Bruker ESP300E spectrometer equipped with a helium flow cryostat (Oxford Instruments, ESR910) and an ITC 503 temperature controller. The X-band spectra were collected with the following parameters: modulation amplitude = 6 G; modulation frequency = 100 kHz; time constant = 20.48 ms; scan time = 167 seconds; single scan. CW Q-band spectra were collected on a Bruker Elexsys E-580 spectrometer at approximately 10 K using CF935 liquid helium cryostat, an ITC-503 temperature controller with a split-ring resonator and the following parameters: modulation amplitude = 6 G; modulation frequency = 100 kHz; time constant = 14.65 ms; scan time = 60 seconds; number of scans = 24 (with no smoothing).

Further X or Q-band pulsed EPR measurements were either collected on a Bruker Elexsys E-580 described above at ~ 5 K or another Bruker Elexsys E-580 equipped with a SuperQ-FT microwave bridge and a closed cycle helium cryostat system (~5 K). The measurement was collected with a slightly overcoupled homebuilt cylindrical TE_{011} resonator. The pulsed EPR spectra were collected with a two-pulse 'Hahn' sequence ($\pi/2$–τ–π–τ–*echo*) where $\pi/2 = 8$ ns for X-band measurements and $\pi/2 = 20$ ns for Q-band measurements; τ was varied and summed in X-band measurements and fixed at 268 ns for Q-band frequency. The pulsed EPR spectrum was collected at 5 K with a repetition time of 9.18 μs.

The EPR spectra were reproduced and simulated with a S = 1/2 spin-Hamiltonian, including both hyperfine coupling from the Sb radical center and two equivalent Ga centers. All EPR spectra were simulated with the EasySpin (ver 5.2) package for Matlab. The mixture of Sb and Ga hyperfine active isotopes at their natural abundance ratios were accounted for within the EPR simulations. The Sb and Ga hyperfine active isotopes exist as: 57.21% ^{121}Sb (I = 5/2) and 42.79% ^{123}Sb (I = 7/2); 60.11% ^{69}Ga (I = 3/2) and 38.89% ^{71}Ga (I = 3/2). The X-band and Q-band spectra were fit simultaneously in a linear-least squares fashion employing a Neadler-Simplex routine to vary the values of g_1, g_2, $A(\mathrm{Sb})_1$, $A(\mathrm{Sb})_2$, $A(\mathrm{Ga}_a)_1$= $A(\mathrm{Ga}_b)_1$ and $A(\mathrm{Ga}_a)_2$=$A(\mathrm{Ga}_b)_2$. The values of g_3, $A(\mathrm{Sb})_3$, and $A(\mathrm{Ga}_a)_3$= $A(\mathrm{Ga}_b)_3$ were determined by manual fitting of the high-field features of each spectra. The high-field edges of both the X- and Q-band spectra of **1** exhibit a resolved hyperfine fine pattern of the g_3 feature. Two isotopes of Sb exist, ^{121}Sb (57.2%, I = 5/2) and ^{123}Sb (42.8%, I = 7/2) each yielding a 6 and 8 line pattern, respectively. Due to nuclear gyromagnetic ratio of the two isotopes (γ_{Sb} = $g_n(^{121}\mathrm{Sb})/g_n(^{123}\mathrm{Sb})$ = $A(^{121}\mathrm{Sb})/A(^{123}\mathrm{Sb})$ = 1.847, the coupling of ^{121}Sb is significantly larger than ^{123}Sb, Therefore, the highest field feature in both the X and Q-band spectra is the $|M_I = +5/2>$ hyperfine manifold of ^{121}Sb. This single feature is further split by two equivalent Ga atoms (^{69}Ga (60.1%, I = 3/2) and ^{71}Ga (38.89%, I = 3/2) (γ_{Ga} = $g_n(^{69}\mathrm{Ga})/g_n(^{71}\mathrm{Ga})$ = 0.787)), to yield a 5 line pattern, allowing for high precision determinination of g_3, and $A_3(\mathrm{Sb})$, $A_3(\mathrm{Ga})$.

Dipolar tensor decomposition.

The simulated hyperfine of Sb yield the absolute values, but do not yield sign information. The isotropic coupling ($a_{iso} = (A_1+A_2+A_3)/3$) must be positive for a radical center, therefore, the principle $A_3(^{121}\mathrm{Sb})$ value is also positive. The unpaired electron is expected to occupy a predominantly p orbital which is anticipated to have axial dipolar character ($\mathbf{T} = \mathbf{A} - a_{iso} = [-t, -t, 2t]$). Most critically, the dipolar tensor yields insight into the axial unpaired spin population, i.e. the p-orbital character. Of the two potential sign assignments for **A** that can yield an approximate axial dipolar **T**, $\mathbf{A}(^{121}\mathrm{Sb})$ = [385, 496, 1138], a_{iso} = 673 MHz or [-385, -496, 1138], a_{iso} = 86 MHz, the later is favored. The small a_{iso} = 86 MHz value indicates negligible unpaired s orbital population at the Sb atom (ρ(Sb s) <0.003) and the value of t = 526 MHz indicates p orbital spin population of ρ(Sb p) = 0.837. This is determined from the isotropic and anisotrpic hyperfine coupling constants of $a_0(^{121}\mathrm{Sb})$ = 35050 MHz and $b_0(^{121}\mathrm{Sb})$ = 628 MHz, respectively. The other hyperfine sign assignment choice of $\mathbf{A}(^{121}\mathrm{Sb})$ = [385, 496, 1138], a_{iso} = 673 MHz, produces s and p orbital unpaired spin population estimates of 0.02 and 0.37, respectively. This is illogical and rejected as it requires more than half of the spin must be delocalized onto the Ga atoms and the ligands, resulting in greater hyperfine coupling constants for Ga than observed.

Magnetic Susceptibility. Solution-state magnetic susceptibilities χ_{M} and effective magnetic moments μ_{eff} of compounds **1** and **4** were determined by ^{1}H NMR spectroscopy using Evans' Method[4] with pure solvent as internal reference and neglecting diamagnetic contributions according to equations (1) and (2).[5] ^{1}H NMR spectra were recorded on a Bruker Avance DPX-300 (^{1}H 300.1 MHz) spectrometer.

$$\chi_M = \frac{3 \cdot \Delta f}{1000 \cdot f \cdot c} \quad (1)$$

$$\mu_{eff} = 798 \cdot \sqrt{T \cdot \chi_M} \quad (2)$$

where χ_M is the molar magnetic susceptibility of the sample in $m^3 \cdot mol^{-1}$, Δf is the chemical shift difference between solvent in presence of paramagnetic solute and pure solvent in Hz, f is the frequency of NMR spectrometer in Hz, c is the concentration of paramagnetic solute in $mol \cdot L^{-1}$, T is the absolute temperature in K, and μ_{eff} is the effective magnetic moment in μ_B.

Magnetic susceptibility data were measured from a powder sample of solid material (**4**) in the temperature range 2 - 300 K by using a SQUID susceptometer (MPMS-7, Quantum Design) with a field of 1.0 T. The experimental data were corrected for underlying diamagnetism by use of tabulated Pascal's constants.[6,7] The simulations with our own package julX were based on the usual spin-Hamilton operator for mononuclear complexes with spin S = 1/2.

Crystallographic methods. Single crystal were obtained from saturated solutions in *n*-hexane at room temperature (**1**, **3**, **LGaClF**), from benzene solution at 8 °C (**2**, **5**) and from toluene solution at -30 °C (**4**), respectively. The crystals were mounted on nylon loops (**1**, **3**, **5**, **LGaClF**) and on glass fibers (**2**, **4**) in inert oil. Data were collected on a Bruker AXS D8 Kappa diffractometer with APEX2 detector (**1**, **3**, **5**, **LGaClF**) and a Stoe IPDS (**2**, **4**) both using monochromated MoKα radiation (λ = 0.71073 Å) at 100(2) K (**1**, **3**, **5**, **LGaClF**), 173(2) K (**2**) and 313(2) K (**4**). The structures were solved by Direct Methods (SHELXS-97) and refined anisotropically by full-matrix least-squares on F^2 (SHELXL-2014).[8] Absorption corrections were performed semi-empirically from equivalent reflections on basis of multi-scans (Bruker AXS APEX2). Hydrogen atoms were refined using a riding model or rigid methyl groups. The bond lengths in the n-hexane molecule of **1** were restrained to be equal (SADI). In **2** H1 was refined freely with its ADP constrained to be 1.5 times the U_{eq} of Sb1 and the Sb-H bond length restrained to be 1.7 Å (DFIX). The bond lengths in the toluene molecules in **4** were restrained to 1.395 and 1.52 Å (DFIX), respectively.

Computational methods. All quantum chemical calculation were employed with the ORCA quantum chemistry package (version 4.0).[9] Ground-state geometry optimizations of **1'**, **4'** and **5'** were calculated with a B3LYP density functional and Def2-QZVP basis set for Bi, Sb, Ga atoms, Def2-TZVP for I, Cl, N atoms, and Def2-SVP for C and H atoms.[10] The RIJCOSX approximation was employed to accelerate the calculations in conjunction with the appropriate auxiliary basis sets (Def2-QZV/J, def2-TZV/J, def2-SV/J).[11] Additionally, effective core potentials (ECP) were employed for Bi, Sb, and I atoms to accelerate geometry optimizations.[12,13] Solvation effects were taken into account by using the conductor-like polarizable continuum model (CPCM), choosing methanol as the solvent in the calculations. Natural bond orbital analysis was performed using the NBO version 6.0.[14]

Supplementary References

[1] Hardman, N. J., Eichler, B. E. & Power, P. P. Synthesis and characterization of the monomer Ga{(NDippCMe)$_2$CH} (Dipp = $C_6H_3Pr^i_2$-2,6): a low valent gallium(I) carbine analogue. *Chem. Commun.* 1991 (2000).

[2] Saleske, H. Ph.D. Dissertation, University of Würzburg, 1983.

[3] Monakhov, K. Y., Zessin, T. & Linti, G. Molecular Assemblies Based on Cp*BiX_2 Units (X = Cl, Br, I): An experimental and Computational Study. *Organometallics* **30**, 2844 (2011).

[4] Evans, D. F. The determination of the paramagnetic susceptibility of substances in solution by nuclear magnetic resonance. *J. Chem. Soc.* 2003 (1959).

[5] Britovsek, G. J. P., Gibson, V. C., Spitzmesser, S. K., Tellmann, K. P., White, A. J. P. & Williams, D. J. Cationic 2,6-bis(imino)pyridine iron and cobalt complexes: synthesis, structures, ethylene polymerization and ethylene/polar monomer co-polymerisation studies. *J. Chem. Soc., Dalton Trans.* 1159 (2002).

[6] O'Connor, C. J. Magnetochemistry - Advances in Theory and Experimentation. *Prog. Inorg. Chem.* **29**, 203 (1982).

[7] Weast, R. C. & Astle, M. J. CRC Handbook of Chemistry and Physics. CRC Press Inc.: Boca Raton, Florida, 1979.

[8] Sheldrick, G. M. Phase annealing in SHELX-90: direct methods for larger structures. *Acta Crystallogr.* **A46**, 467 (1990); Sheldrick, G. M. SHELXL-2014, Program for the Refinement of Crystal Structures University of Göttingen, Göttingen (Germany) 2014; Sheldrick, G. M. A short history of SHELX. *Acta Crystallogr.* **A64**, 112 (2008); Hübschle, C. B., Sheldrick, G. M. & Dittrich, B. ShelXle: a Qt graphical user interface for SHELXL. *J. Appl. Cryst.* **44**, 1281 (2011).

[9] Neese, F. The ORCA program system. *Wiley Interdisciplinary Reviews: Computational Molecular Science* **2**, 73 (2012).

[10] Weigend, F. & Ahlrichs, R. Balanced basis sets of split valence, triple zeta valence and quadruple zeta valence quality for H to Rn: Design and assessment of accuracy. *Phys. Chem. Chem. Phys.* **7**, 3297 (2005).

[11] Weigend, F. Accurate Coulomb-fitting basis sets for H to Rn. *Phys. Chem. Chem. Phys.* **8**, 1057 (2006).

[12] Metz, B., Stoll, H. & Dolg, M. Small-core multiconfiguration-Dirac-Hartree-Fock-adjusted pseudopotentials for post-d main group elements: Application to PbH and PbO. *J. Chem. Phys.* **113**, 2563 (2003).

[13] Peterson, K. A., Figgen, D., Goll, E., Stoll, H. & Dolg, M. Systematically convergent basis sets with relativistic pseudopotentials. II. Small-core pseudopotentials and correlation consistent basis sets for the post-d group 16-18 elements. *J. Chem. Phys.* **119**, 11113 (2003).

[14] NBO 6.0: Glendening, E. D., Badenhoop, J. K., Reed, A. E., Carpenter, J. E., Bohmann, J. A., Morales, C. M., Landis, C. R. & Weinhold F. (Theoretical Chemistry Institute, University of Wisconsin, Madison, WI, 2013); http://nbo6.chem.wisc.edu/.
